# Supplementary material for: Astrocytes Resist HIV-1 Fusion but Engulf Infected Macrophage Material
Source: Cell Rep. 2017 Feb 7;18(6):1473–83. doi: 10.1016/j.celrep.2017.01.027 (PMC5316642; doi:10.1016/j.celrep.2017.01.027)
Supplement: Document S1. Supplemental Experimental Procedures and Figures S1–S5 [file mmc1.pdf]

Cell Reports, Volume *18*

Supplemental Information

Astrocytes Resist HIV-1 Fusion but Engulf Infected Macrophage Material

Rebecca A. Russell, Jakub Chojnacki, Daniel M. Jones, Errin Johnson, Thao Do, Christian Eggeling, Sergi Padilla-Parra, and Quentin J. Sattentau

Supplementary materials for Russell et al "Astrocytes resist HIV-1 fusion but engulf infected macrophage material"

Supplementary methods

Viruses

Replication-competent infectious molecular clones stably expressing either Renilla luciferase or GFP in an isogenic NL4.3 backbone (NL-LucR.T2A) encoding HIV-1 BaL, HIV-1 YU2 and HIV-1 NL4.3 *env* ectodomains in *cis* (Ochsenbauer et al., 2012) were prepared by transfection using polyethyleneimine (PEI, Sigma-Aldrich) in HEK-293T cells and titered on TZM-bl cells. Single cycle pseudovirus vectors were prepared by transfecting 293T cells with the vector backbone pNL4.3.Luc.R-E- (NIH AIDS Research and Reference Reagent Program) (He et al 1995), pMM310 (NIH AIDS Research and Reference Reagent Program) (Tobiume et al, 2003) expressing the Vpr-β-lactamase fusion protein and one of the following Env-expressing plasmids; pHEF-VSV-G (NIH AIDS Research and Reference Reagent Program)(Chang L-J, et al. 1999), BaL, JRFL and LAI (NIH AIDS Research and Reference Reagent Program). Pseudovirus vectors were titered on TZM-bl cells and titer determined using ID-50 software version 5.0. For the live cell BlaM assay, cells were transfected with 2µg pR8ΔEnv, 2µg Vpr-BlaM, 1µg pcREV and 3µg of the appropriate viral envelope (either VSV-G or the CCR5-tropic HIV-1 strain JR-FL) using GeneJuice (Novagen). At 12 hr post-transfection the transfection mixture-containing medium was removed, cells washed with PBS and fresh $DMEM_{comp}$ (lacking phenol red) added and cells incubated for a further 24 hr. At 48 hr post-transfection viral supernatants were harvested, aliquoted and stored at -80˚C. For single virus tracking, cells were transfected in the same manner with the following modification: at 12 hr post-transfection cells were washed with PBS then incubated with 10ml Optimem (Life Technologies) containing 10µM DiD (Life Technologies) for 4 hr. The staining mixture was removed, cells washed twice with PBS and fresh $DMEM_{comp}$ (lacking phenol red) added, and cells incubated for a further 24 hr prior to harvesting.

Flow cytometry

Cells were prepared for surface labeling flow cytometry using fluorochrome-conjugated monoclonal antibody (mAb) labeling and matching isotype controls. MAbs used in experiments were: CD4-APC (clone RPA-T4 Becton Dickinson, BD); CXCR4-APC (clone 12G5 Biolegend); CCR5 (clone HEK/1/85a Biolegend). Cells

were pelleted in a 96 well round bottom plate and incubated in PBS with 2% FBS/0.01% sodium azide (wash buffer, WB), 10 µg/mL normal human IgG and conjugated antibody for 1 hr at 4^0C. Cells were washed with in WB and fixed with 4% formaldehyde in PBS at RT for 15 mins. Cells were washed 3 times with PBS and re-suspended in PBS for analysis. For labeling of fixed cells, cells were fixed in 4% formaldehyde in PBS prior to staining, as described above. For labeling intracellular markers cells were fixed as above and permeabilized in WB containing 0.1% saponin prior to staining, as described above. Data from 5-10 x 10^3 events per sample were acquired using a FACS Calibur (BD). Analysis was with FlowJo V10 software.

BlaM-Vpr fusion assay

HFA and MDM, seeded in 12-well plates at $1x10^6$ and $7.5x10^4$ cells/well respectively, were spinoculated at 1200 x g at 37°C for 45 min with 800 IU of BlaM-Vpr-containing PV expressing BaL, LAI or VSV-G Env. Supernatant was removed and replaced with CCF2 prepared in CO_2-independent medium (CO_2-IM) (Life technologies) and cells incubated for 1 hr at room temerapture (RT) in the dark. Cells were washed 3 times in CO_2-IM and incubated for 16 hr at RT in the dark in CO_2-IM / 10% FBS / 2.5 mM probenecid (Sigma). Cells were lifted using trypsin (Life technologies), fixed and analyzed on a CyAn ADP (Dako). In drug treatment experiments, cells (or virus for T20), were pretreated for 1 hr at 37°C (or RT for T20), prior to spinoculation. Drug supernatant concentration was maintained at every stage of the assay.

Real time BLaM-Vpr assay image acquisition and analysis

Cells loaded with CCF2 were excited using a 405 nm continuous laser (Leica, Manheim) and the emission spectra between 430-560 nm recorded pixel by pixel (512 X 512) with a lambda resolution of 12 nm. The ratio of blue emission (440 - 480 nm, cleaved CCF2) to green (500 - 540 nm, un-cleaved CCF2) was calculated pixel by pixel using ImageJ (http://imagej.nih.gov/ij/) for three different 20 X objective observation fields in each well and plotted as a function of time. A blue/green threshold (fusion threshold) was set using Env-negative ($HIV_{No\ Env}$) virions containing BlaM-Vpr as a background control to provide a fusion detection limit that corresponded to 0.3+/- 0.05 BlaM ratio. The fusion threshold was calculated by recovering the signal (blue/green intensity ratio) coming from individual cells at the last time point (~100 min for MDM and ~140 min for HFA) plus 2 x SD from ~300 cells in the observation field. This threshold was then applied to all images at all time points of cells treated with $HIV_{No\ Env}$, $HIV_{VSV\text{-}G}$ and $HIV_{JR\text{-}FL}$. Cells above the threshold

where pseudocolored in red while cells below the threshold where pseudocolored in blue. "Red" cells were then counted as a function of time to recover the number of fusogenic cells that corresponds to an accurate measure of viral fusion kinetics.

Live cell single particle image acquisition and analysis

Cells were plated at $3x10^5$ (MDM) or $1x10^5$ (HFA) cells/well in 8-well µ-Slides (Ibidi) and double-labelled virions (Gag-iGFP and DiD) were incubated for 30 min at 4 °C and cells washed with PBS to remove unbound virions. Cells were mounted onto the confocal Leica SP8XSMD with a stage maintained at 37 °C. Once a suitable image field was chosen, virus internalization was initiated by adding 1 mL of warm HBSS and imaging commenced using a Leica SP8 confocal microscope with a 63×/1.4 NA oil immersion objective and 2 HyD internal detectors capable of single photon counting. Slow loss of red and green signals over time reflects particle drift outside the plane of focus and some photobleaching in the green channel. True fusion events are represented by strict criteria (Jones and Padilla-Parra, 2015; Padilla-Parra et al., 2013; Padilla-Parra et al., 2014) defined by a rapid (~1 min) drop in either the green signal whilst the red color (DiD) remains stable (endosomal fusion). Images were acquired every 8–12 s for ~45 min. The axial position of a specimen during acquisition was stabilized using the Adaptative Focus module. GFP was excited at 488 nm and emitted light was collected at 500–550 nm. DiD was excited at 633 nm and the emission range was 640–700 nm. These imaging conditions ensured negligible bleed-through between GFP and DiD channels. Virus particles that entered the cell were tracked using the red channel as a reference. Real time single particle tracking analyses yielded the mean fluorescence intensity of viral particles for both red and green channels, and their coordinates, as a function of time. SVT analysis was performed using Imaris software (BitPlane, Switzerland).

Transmission electron microscopy

Cells were cultured on sterilized glass coverslips, fixed with 2.5% gluteraldehyde in 0.1 M Sodium Cacodylate Buffer at pH 7.2 or 0.1M PBS for 1 hr at RT then rinsed in sodium Cacodylate buffer 3 times, 15 mins each. Samples were stained in 1% osmium tetroxide in sodium Cacodylate buffer at RT for 1 h, rinsed with fresh Milli-Q water 3 times, 5 mins each, then stained with 2% uranyl acetate in Milli-Q water for 2 h at 4°C protected from light. The samples were rinsed with water for 10 mins, dehydrated in an ethanol series, embedded in resin and prepared for microscopy. Images were acquired on an FEI Tecnai 12 Transmission Electron Microscope

(TEM) with a 4 Megapixel Gatan Ultrascan™ 1000 CCD camera. Images were taken at 100-100,000x magnification using Gatan Digital Micrograph software at 120 kV using a lanthanum hexaboride (LaB6) electron source and analyzed using ImageJ software.

Confocal and STED microscopy image acquisition, processing and analysis

The microscope used for both confocal and STED images was based on an Abberior Instrument RESOLFT QUAD-P super-resolution microscope (Abberior Instruments) additionally equipped with two pulsed excitation lasers: 640 nm (LDH-D-C-640P, PicoQuant) and 485 nm (LDH-D-C-485P, PicoQuant), both with an 80 ps pulse width. The STED beam was produced by a titanium:sapphire laser system (MaiTai, Spectra-Physics) operating at 780 nm with a repetition rate of 80 MHz. The STED laser pulses were stretched to a pulse width of approximately 250-350 ps using two 20 cm SF6 optical glass rods, and a 121 m long polarization-maintaining single-mode optical fibre (OZ Optics). A phase-modifying plate (VPP-1a, RPC Photonics) was used in the STED beam path to generate a donut-shaped focal spot. STED and excitation laser beams were spatially superimposed and the fluorescence light was filtered using appropriate dichroic filters (AHF Analysentechnik). Precise positioning of the laser foci in the sample, and sample scanning was carried out using a QUAD beam scanner for lateral directions and an objective lens positioning system (MIPOS 100PL, Piezosystem Jena) for axial direction. The pulse timing was adjusted using a combination of an electronic delay box (Picosecond Delayer, Micro Photon Devices) and cables. Fluorescence excitation and collection used an oil immersion 100x, 1.40 NA objective (Olympus). The fluorescence signal was de-scanned, passed through an adjustable pinhole (Thorlabs) and detected by a single photon counting avalanche photo diode (SPCM-AQRH-13, Excelitas Techologies) with appropriate fluorescence filters. Data were acquired using Imspector™ software.

For confocal microscopy, images for each channel were acquired sequentially with following parameters: pinhole size: 1 Airy, *xy* pixel size: 100 nm, *z*-step size: 250 nm STED images were acquired sequentially for each channel with following parameters: pinhole size: 1 Airy, dwell time: 50 µs/pixel, *xy* pixel size: 40 nm, *z*-step size: 250 nm.

For STED microscopy images for each channel were acquired sequentially with following parameters: pinhole size: 1 Airy; dwell time: 50 µs/pixel, XY pixel size: 40 nm, Z-step size: 250 nm. STED images were deconvoluted using Huygens

Professional software using theoretical PSF parameters corresponding to the system's effective observation spot or point-spread-function (PSF, 50 nm in diameter, full-width-half-maximum of intensity profile) as confirmed by measurements of 20 nm fluorescent beads. All subsequent image manipulation steps were performed using Fiji (ImageJ distribution) software package. Minimal distance between HIV-1 particles and HFA plasma membrane was calculated using GFAP and HIV p24 fluorescent signals as guides. Due to the confocal only resolution in the *z* direction making accurate positioning difficult, distances were calculated in the *xy* plane only. Images in *z*-stack were analyzed separately under assumption that each virus particle is localized in a *z*-slice with the brightest p24 fluorescence signal. Images with a significant number of particles localized directly above or below the HFA membrane were omitted. Env content of HFA internalized and cell-free HIV-1 particles was calculated as follows. The percentage of Env^+ HIV-1 particles inside HFA was calculated by comparing the percentage of Env^+ HIV-1 particles inside HFA with cell free particles present within the same slide, using GFAP, p24 and gp120 fluorescence signals as guides.

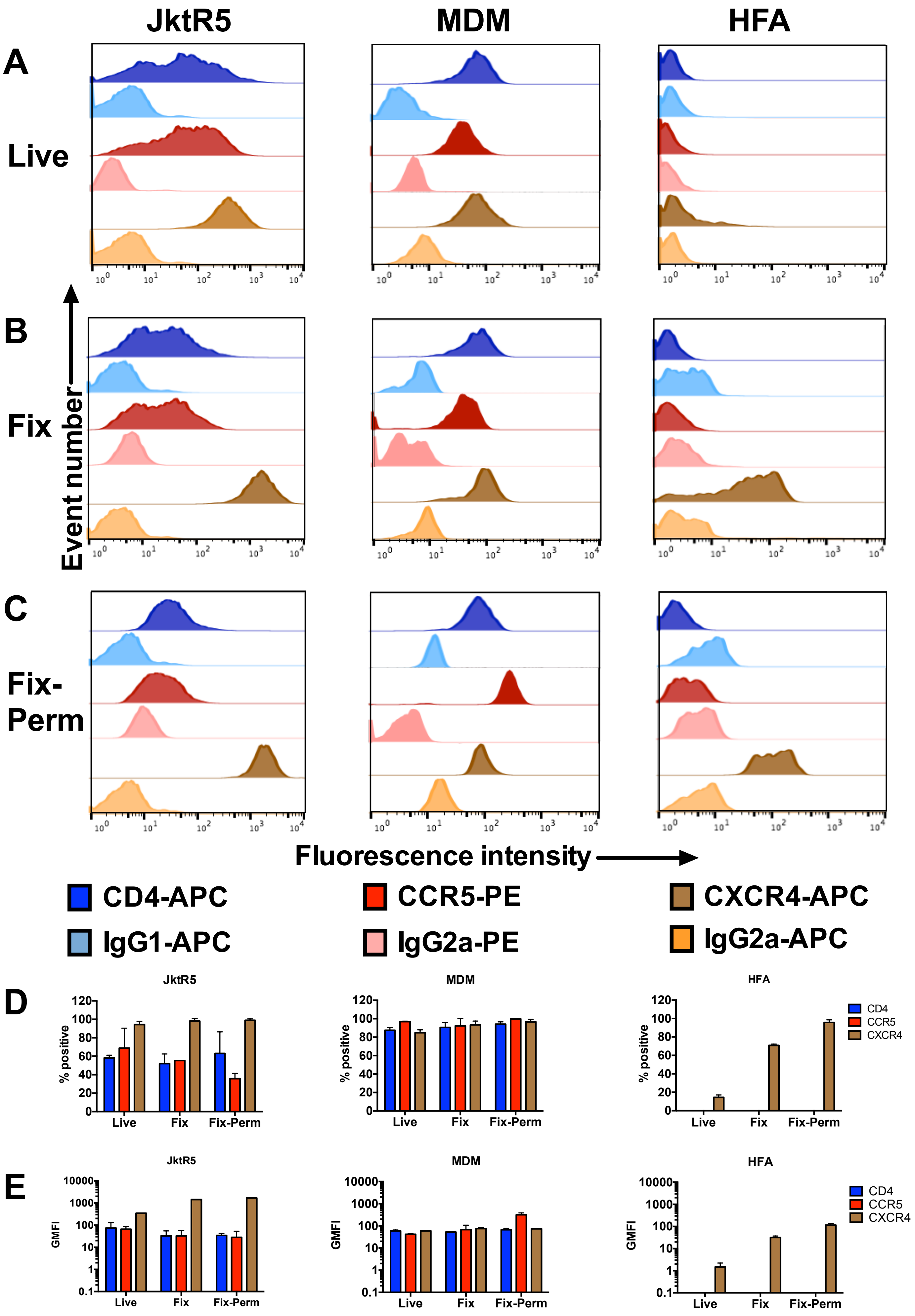
JktR5
MDM
HFA
A
Live
B
Fix
C
Fix-
Perm
Event number
Fluorescence intensity
CD4-APC
IgG1-APC
CCR5-PE
IgG2a-PE
CXCR4-APC
IgG2a-APC
D
% positive
Live
Fix
Fix-Perm
CD4
CCR5
CXCR4
E
GMFI

Supplementary Figure 1. Flow cytometric analysis of HFA phenotype. A) Jurkat-R5 (Jkt-R5), monocyte-derived macrophages (MDM) and HFA were labeled live for CD4 (dark blue) or isotype control (light blue), CCR5 (red) or isotype control (pink) and CXCR4 (brown) or isotype control (orange) and analyzed by flow cytometry. **B)** Jkt-R5, MDM and HFA were fixed (Fix) and labeled as in **(A)**. **C)** Jkt-R5, MDM and HFA were fixed, permeabilized (Fix-Perm) and labeled as in **(A)**. **D)** Summary of data from **(A-C)** showing % positive cells, n = 2 independent experiments with 2 independent macrophage donors. **E)** Summary of data from **(A-C)** showing geometric mean fluorescence intensity (GMFI) , n = 2 independent experiments with 2 independent macrophage donors.

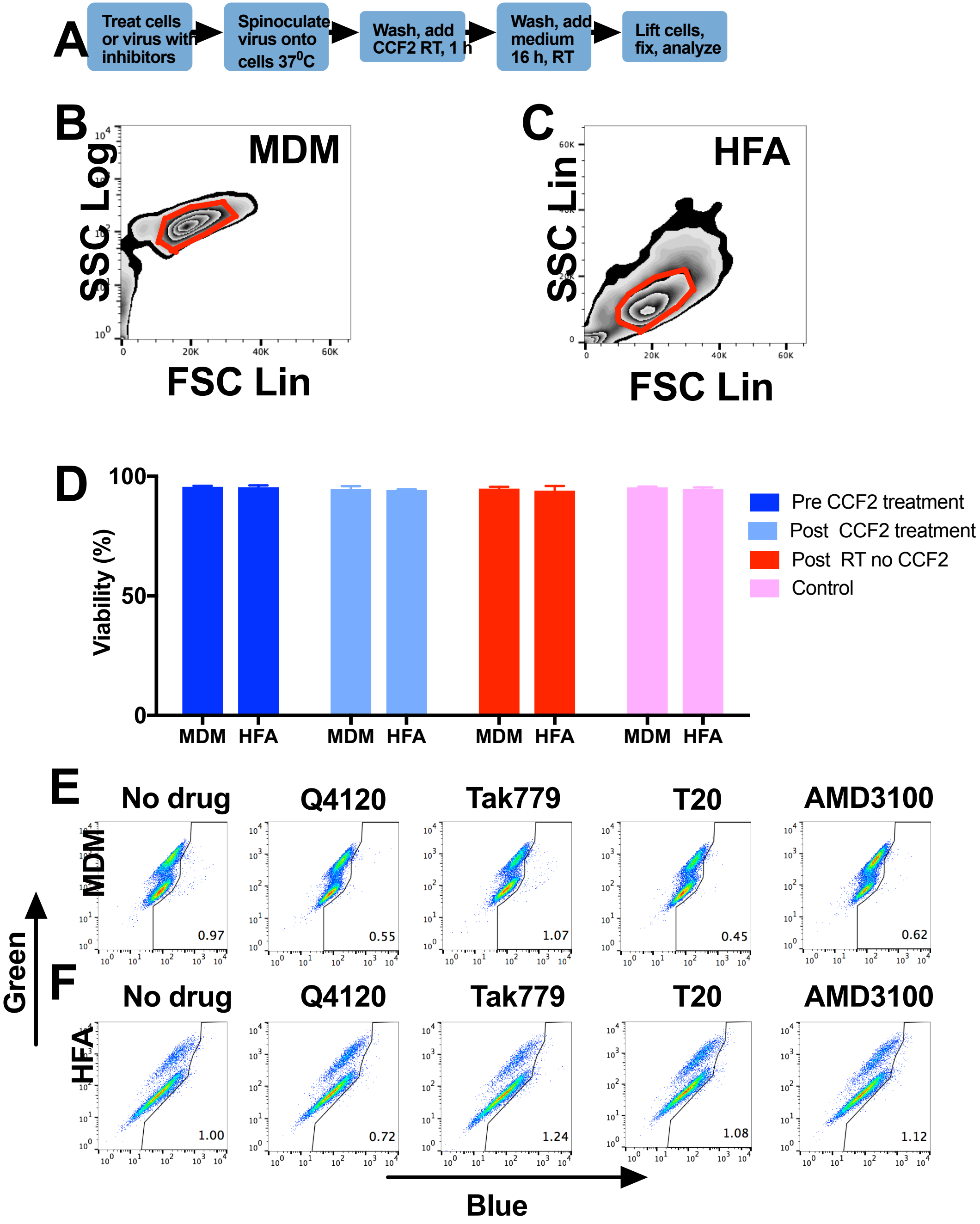

Supplementary Figure 2. HIV-1 LAI pseudotypes do not fuse with HFA, related to Figure 2. A) Experimental flow plan. **B)** Representative forward scatter (FSC) verses side scatter (SSC) of MDM after viral exposure and CCF2 treatment. Red region shows area selected for analysis in **E** and Figure 2. **C)** Representative FSC verses SSC of HFA after viral exposure and CCF2 treatment. Red region shows area selected for analysis in **F** and Figure 2. **D)** Cell viability before (Pre CCF2 treatment) and after CCF2 exposure (Post CCF2 treatment) compared to cells maintained at room temperature in the absence of CCF2 (Post RT no CCF2) and cells maintained at 37^{0}C with 5% CO_2 (Control), as determined by trypan blue, n = 1 independent experiment with technical triplicates. **E)** MDM were untreated or treated with inhibitors then transduced with LAI-pseudotyped BLaM-Vpr HIV-1 for 16 hr then cells lifted, fixed and analyzed by flow cytometry. **F)** HFA were untreated or treated with inhibitors then with LAI-pseudotyped BLaM-Vpr HIV-1 for 16 hr then cells lifted, fixed and analyzed by flow cytometry. See Figure 2H for quantified summary of data.

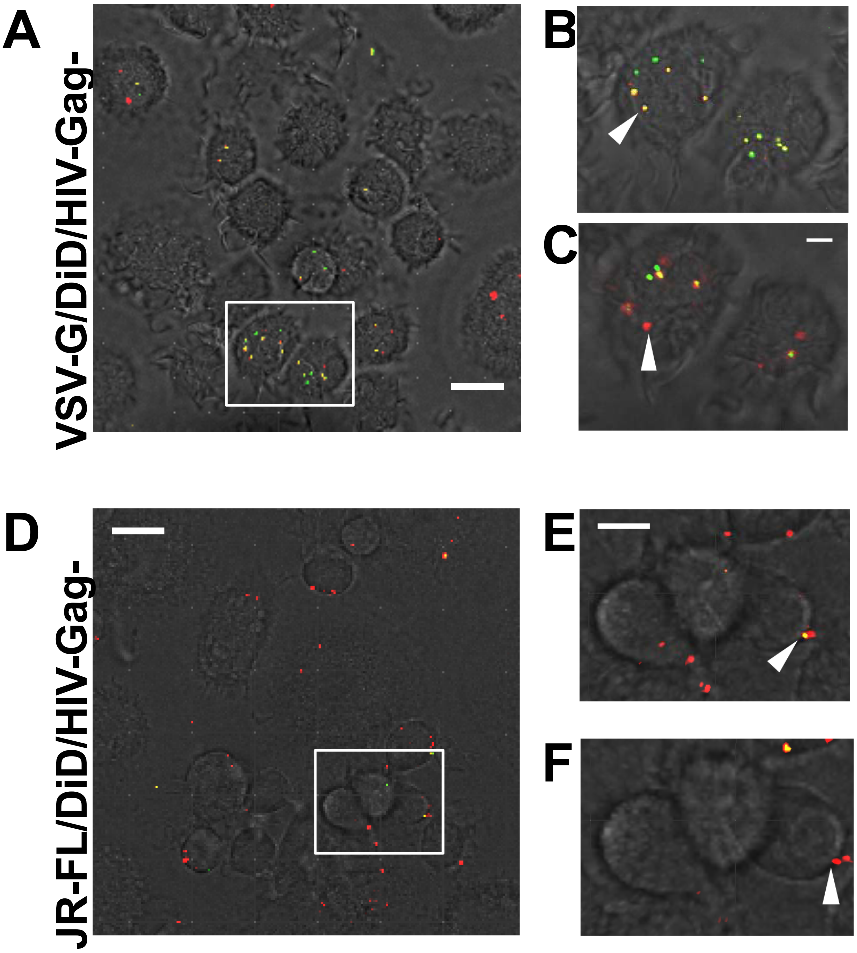

Supplementary Figure 3. Real-time single virus tracking reveals JR-FL and VSV-G-psuedotyped HIV-1 fusion with MDM, related to Figure 4. A) A representative experiment of particles double-labeled with DiD and Gag-iGFP, pseudotyped with VSV-G bound to live MDM. Scale bar 20 µm. **B** and **C)** Snap-shots of micrographs taken from the boxed section in **A** at 0 and 5 min, respectively. The yellow particle (white arrow, **B**) tracked over 5 min becomes red in **C** (white arrow). Scale bar: 2 µm. **D)** A representative experiment of particles double-labeled with DiD and Gag-iGFP, pseudotyped with JRFL bound to live MDM. Scale bar 20 µm. **E** and **F**) Snap-shots of micrographs taken from the boxed section in the **D** at 0 and 5 min, respectively. The yellow particle (white arrow, **E**) undergoes fusion and turns red (white arrow **F**). Scale bar: 4 µm. N = 3 independent experiments on 3 independent macrophage donors.

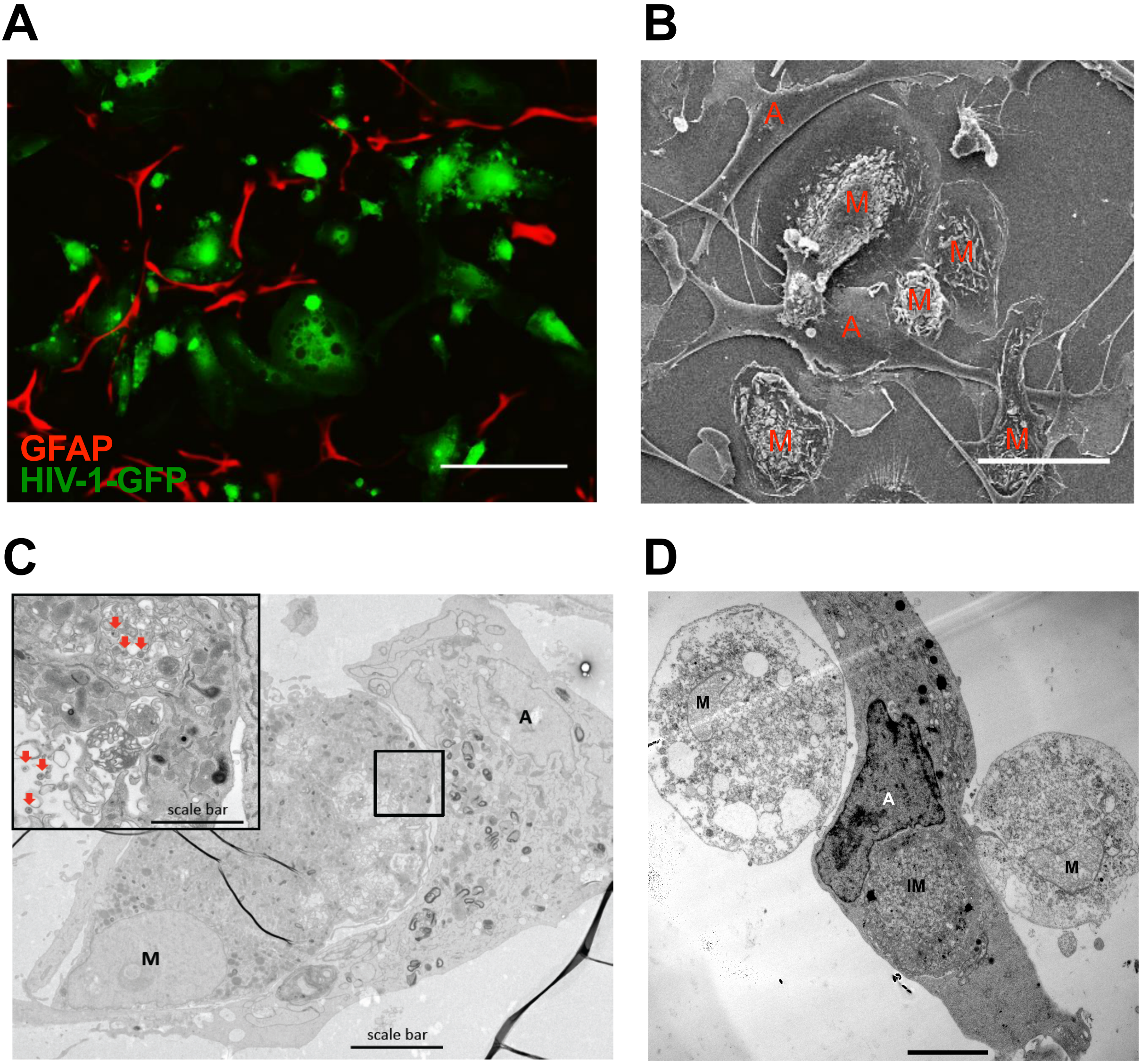

Supplementary Figure 4. Intimate associations form between HFA and HIV-1-infected MDM, related to Figures 5 and 6. A) Cocultures of HIV-1$_{JR\text{-}FL\text{-}GFP}$-infected MDM and GFAP-labeled HFA imaged by confocal microscopy, scale bar = 50 µm. **B)** SEM image of an MDM-HFA coculture showing MDM (M) interacting with HFA (A), scale bar = 25 µm. **C)** TEM image of HIV-1-infected MDM (M, labeled over nucleus) partially engulfed by an astrocyte (A. labeled over nucleus). Scale bar = 5 µm. Inset box shows higher magnification image, red arrows point to virions. Scale bar = 1 µm. **D)** TEM image of three MDM (M) interacting with HFA (A). IM = internalized macrophage. M and A label nuclei of cells. Scale bar = 5 µm

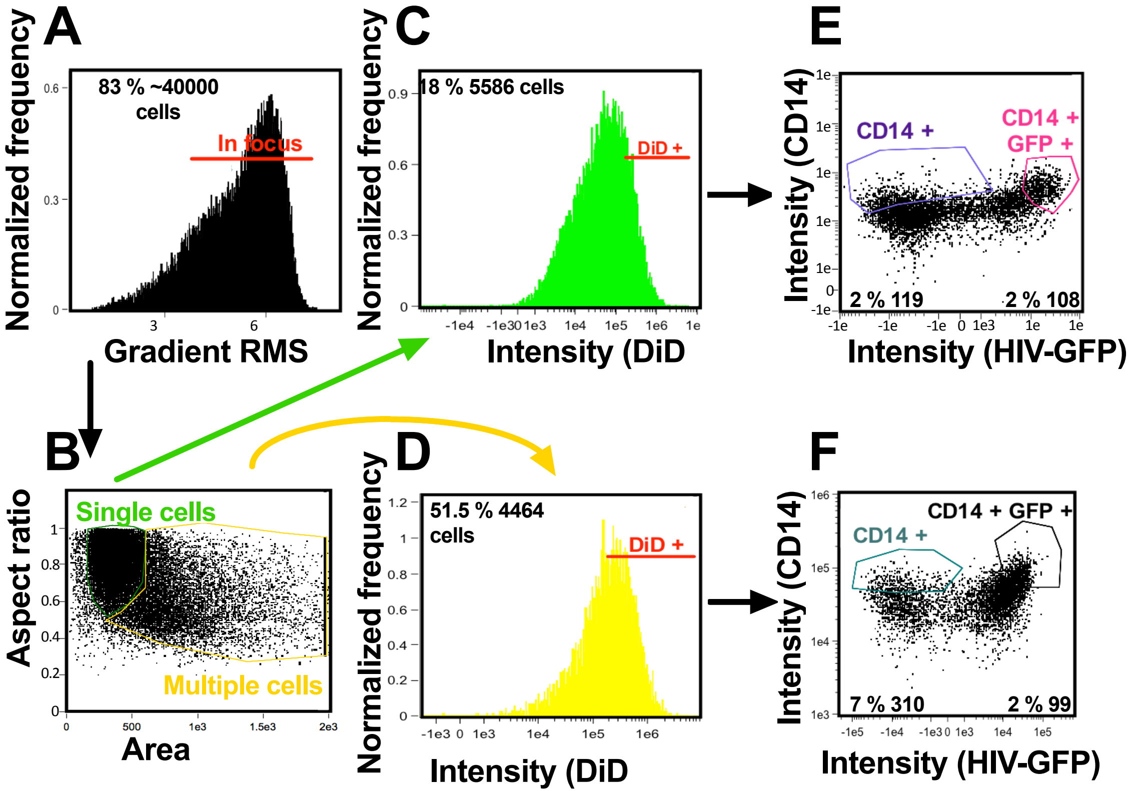

Supplementary Figure 5. Imagestream analysis of interactions between HFA and HIV-1-infected MDM, related to Figure 6. Gating strategy for image stream **A)** Selection of in focus cells. **B)** Selection of single and multiple cells. **C)** Selection of DiD^+ single cells. **D)** Selection of DiD^+ multiple cells. **E)** Selection of $CD14^+$ and $CD14^+/GFP^+$ cells within the DiD^+ single cells. **F)** Selection of $CD14^+$ and $CD14^+/GFP^+$ cells within the DiD^+ multiple cells.

Supplementary Movie 1. Real-time single virus tracking shows VSV-G-pseudotyped HIV-1 particles fusing with HFA related to Figure 4. VSV-G-psuedotyped HIV-1 virions double-labeled with Gag-iGFP (green signal) and DiD (red signal) were imaged in real time on live HFA plated in observation chambers under a confocal microscope. Double labeled virions (yellow) were tracked and fusion defined by the change from yellow to red (indicated by two white arrows). The movie plays at 12 frames / s and total duration is ~15 min.

Supplementary Movie 2. Real-time single virus tracking shows JRFL-pseudotyped HIV-1 particles fail to fuse with HFA, related to Figure 4. JRFL-pseudotyped HIV-1 virions double-labeled with Gag-iGFP (green signal) and DiD (red signal) were imaged in real time on live HFA plated in observation chambers under a confocal microscope. Double labeled virions (yellow) were tracked and failed to show fusion as defined by the change from yellow to red. Loss-of-focus and slight bleaching is observed in some of the particles over time. The movie plays at 12 frames / s and total duration is ~40 min.

Supplementary Movie 3. Real-time single virus tracking shows VSV-G-pseudotyped HIV-1 particles fuse with MDM, related to Figure S3. VSV-G-pseudotyped HIV-1 virions double-labeled with Gag-iGFP (green signal) and DiD (red signal) were imaged in real time on live MDM plated in observation chambers under a confocal microscope. A double labeled virion (yellow) was tracked and fusion defined by the change from yellow to red (indicated by white arrow). The movie plays at 12 frames / s and total and duration is ~7 min.

Supplementary Movie 4. Real-time single virus tracking shows JRFL-pseudotyped HIV-1 particles fuse with MDM, related to Figure S3. JRFL-pseudotyped HIV-1 virions double-labeled with Gag-iGFP (green signal) and DiD (red signal) were imaged in real time on live HFA plated in observation chambers under a confocal microscope. A double labeled virion (yellow) was tracked and fusion defined by the change from yellow to red (indicated by white arrow). The movie plays at 12 frames / s and total duration is ~10 min.

Supplementary Movie 5. Dynamic rendering of static images presented in Figure 6H, I.

Supplementary Movie 6. Dynamic rendering of static images presented in Figure 6J, K.
