# Supplementary figures and images for: Astrocytes Resist HIV-1 Fusion but Engulf Infected Macrophage Material
Source: Cell Rep. 2017 Feb 7;18(6):1473–83. doi: 10.1016/j.celrep.2017.01.027 (PMC5316642; doi:10.1016/j.celrep.2017.01.027)

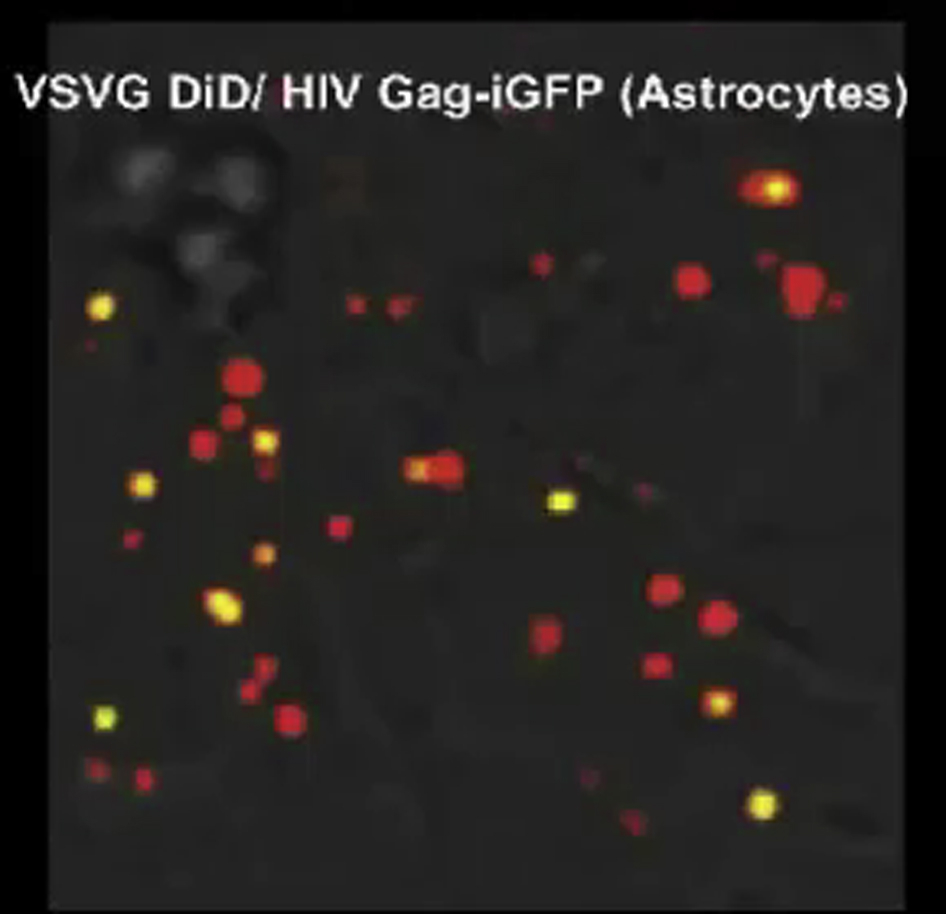

Supplement: Movie S1. Real-Time Single-Virus Tracking Shows VSV-G-Pseudotyped HIV-1 Particles Fusing with HFAs, Related to Figure 4 [file mmc2.jpg]

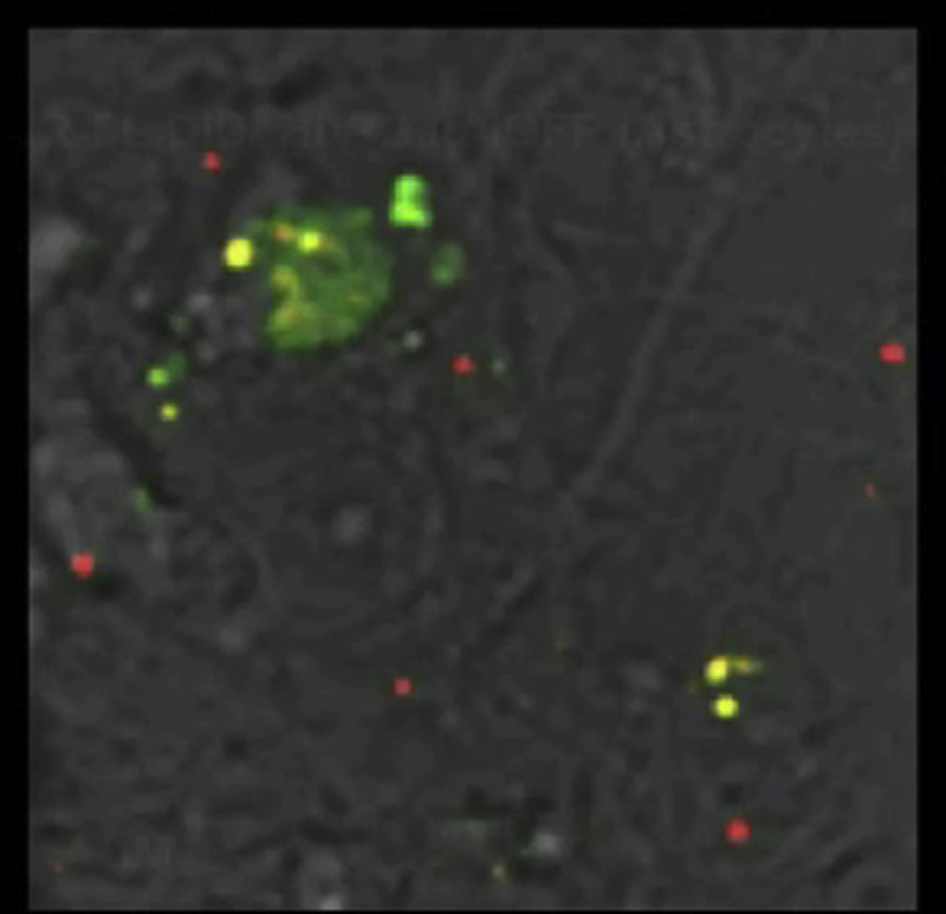

Supplement: Movie S2. Real-Time Single-Virus Tracking Shows JRFL-Pseudotyped HIV-1 Particles Fail to Fuse with HFAs, Related to Figure 4 [file mmc3.jpg]

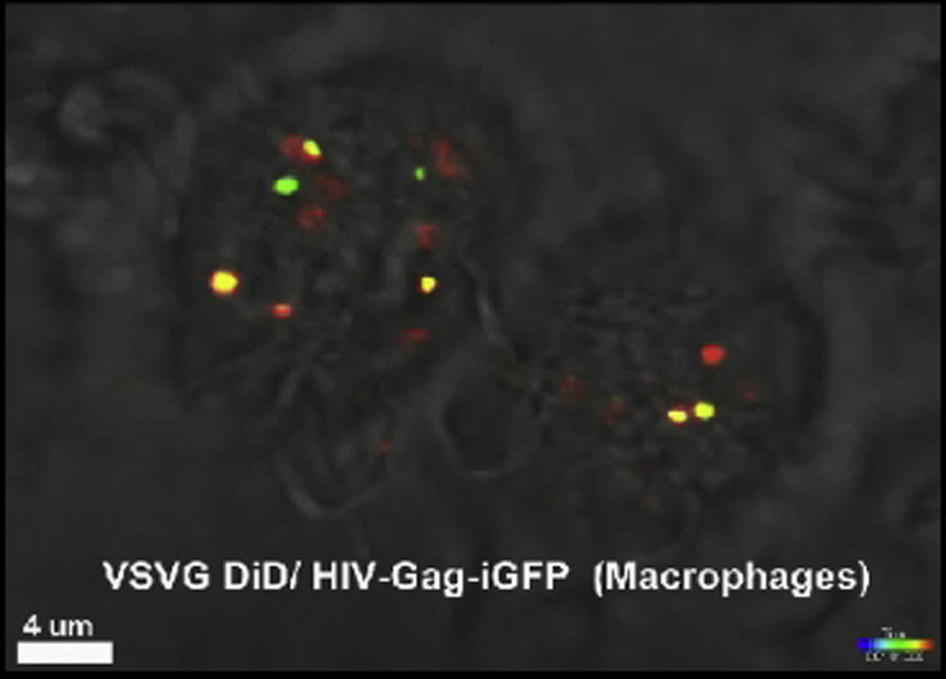

Supplement: Movie S3. Real-Time Single-Virus Tracking Shows VSV-G-Pseudotyped HIV-1 Particles Fuse with MDMs, Related to Figure S3 [file mmc4.jpg]

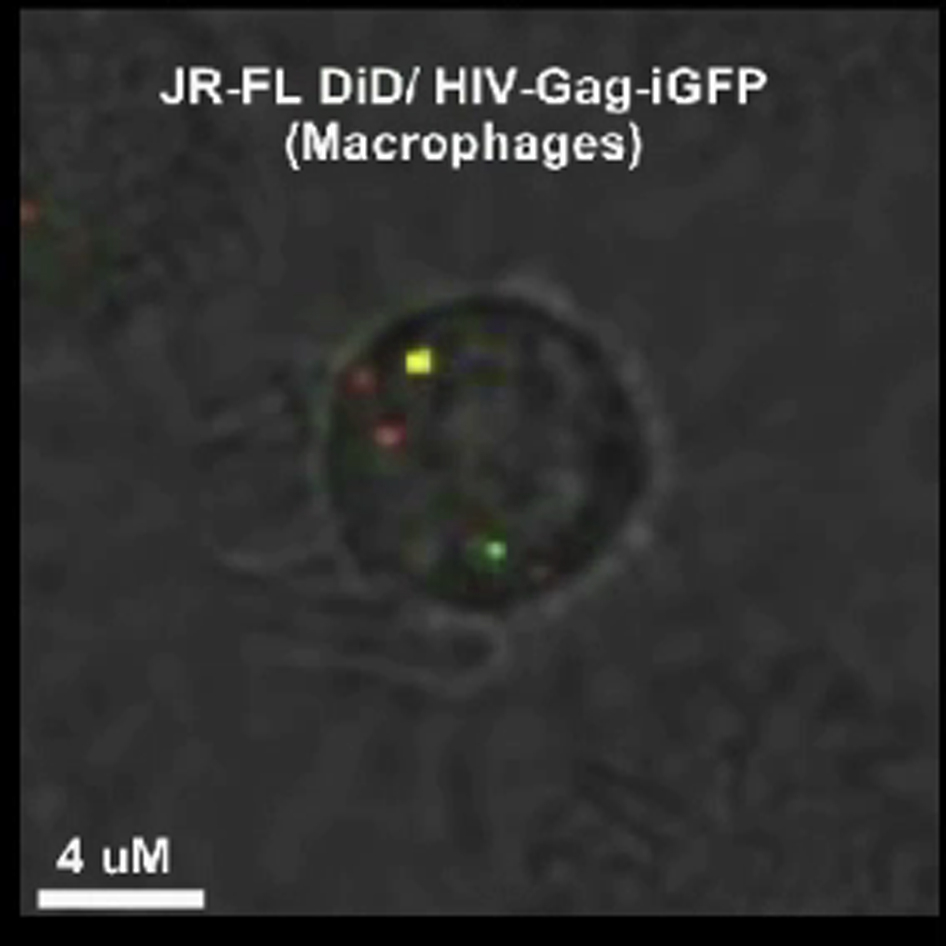

Supplement: Movie S4. Real-Time Single-Virus Tracking Shows JRFL-Pseudotyped HIV-1 Particles Fuse with MDMs, Related to Figure S3 [file mmc5.jpg]

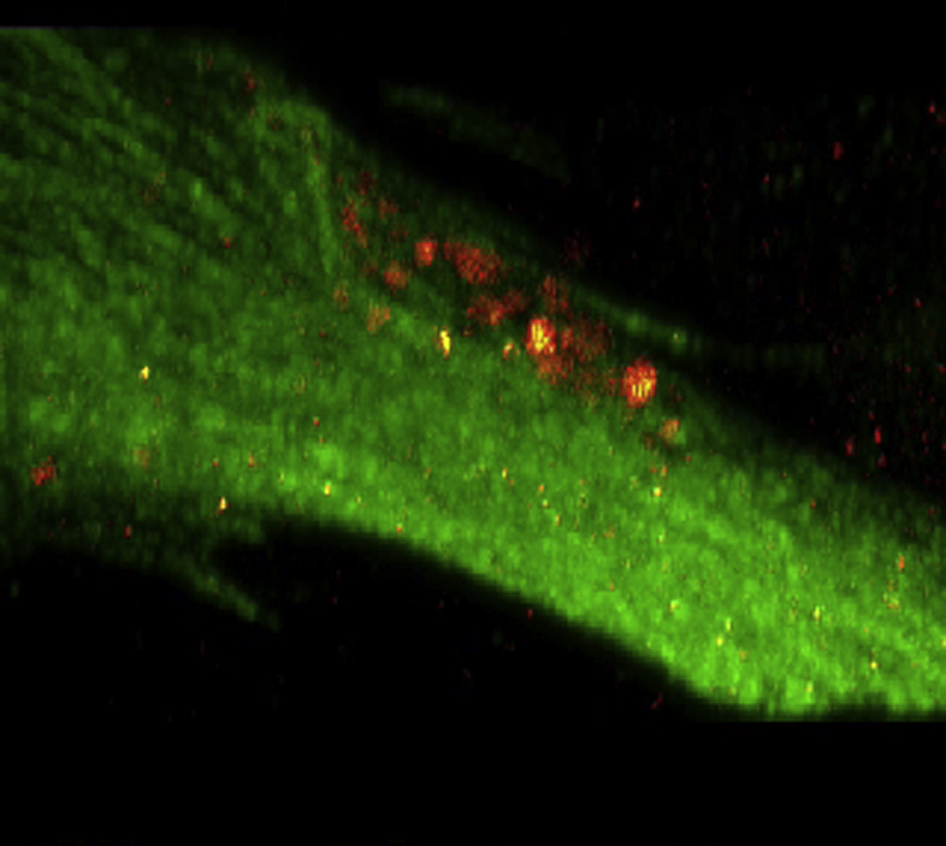

Supplement: Movie S5. Dynamic Rendering of Static Images Presented in Figures 6H and 6I [file mmc6.jpg]

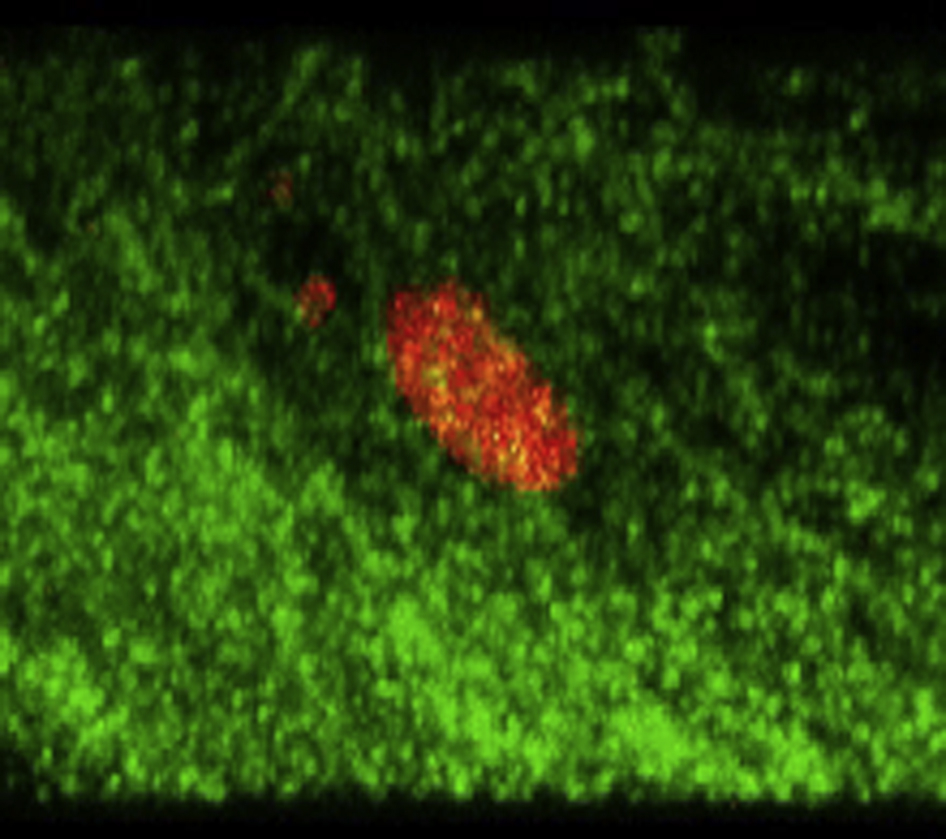

Supplement: Movie S6. Dynamic Rendering of Static Images Presented in Figures 6J and 6K [file mmc7.jpg]
