# Supplementary material for: Astrocytes Resist HIV-1 Fusion but Engulf Infected Macrophage Material
Source: Cell Rep. 2017 Feb 7;18(6):1473–83. doi: 10.1016/j.celrep.2017.01.027 (PMC5316642; doi:10.1016/j.celrep.2017.01.027)
Supplement: Document S2. Article plus Supplemental Information [file mmc8.pdf]

Cell Reports

Article

Astrocytes Resist HIV-1 Fusion but Engulf Infected Macrophage Material

Graphical Abstract

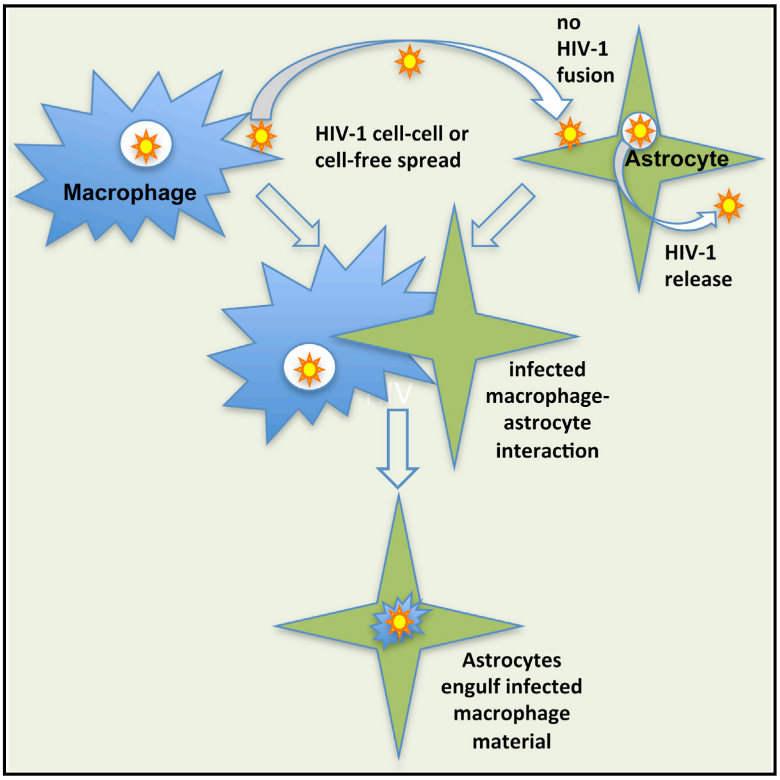

Highlights

- Astrocytes may endocytose virions from cell-free or cell-cell sources
- HIV-1 virions do not fuse with astrocytes and astrocytes are not infected
- Astrocytes cluster with HIV-1-infected macrophages
- Astrocytes engulf macrophage material, including HIV-1-infected debris

Authors

Rebecca A. Russell, Jakub Chojnacki, Daniel M. Jones, ..., Christian Eggeling, Sergi Padilla-Parra, Quentin J. Sattentau

Correspondence

quentin.sattentau@path.ox.ac.uk

In Brief

HIV-1 disseminates to diverse tissues and establishes long-lived viral reservoirs. These reservoirs include macrophage-lineage cells in the CNS, and previous studies indicate that astrocytes may be infected. Russell et al. find that astrocytes are not infected by virus but can engulf macrophage material, including HIV-1-infected debris.

Russell et al., 2017, Cell Reports *18*, 1473–1483
February 7, 2017 © 2017 The Author(s).
http://dx.doi.org/10.1016/j.celrep.2017.01.027

Cell Reports
Article

OPEN ACCESS
CellPress

Astrocytes Resist HIV-1 Fusion but Engulf Infected Macrophage Material

Rebecca A. Russell,[1,6] Jakub Chojnacki,[2,6] Daniel M. Jones,[3,4,6] Errin Johnson,[1] Thao Do,[5] Christian Eggeling,[2] Sergi Padilla-Parra,[3,4] and Quentin J. Sattentau[1,7,*]

[1]The Sir William Dunn School of Pathology, University of Oxford, Oxford OX1 3RE, UK
[2]MRC Human Immunology Unit, Weatherall Institute of Molecular Medicine, University of Oxford, Oxford OX3 9DS, UK
[3]Division of Structural Biology, University of Oxford, The Henry Wellcome Building for Genomic Medicine, Headington, Oxford OX3 7BN, UK
[4]Wellcome Trust Centre for Human Genetics, Cellular Imaging Core, University of Oxford, Oxford OX3 7BN, UK
[5]Laboratory of Cell Biology, Center for Cancer Research, National Cancer Institute, National Institutes of Health, Bethesda, MD 20892, USA
[6]Co-first author
[7]Lead Contact
*Correspondence: quentin.sattentau@path.ox.ac.uk
http://dx.doi.org/10.1016/j.celrep.2017.01.027

SUMMARY

HIV-1 disseminates to diverse tissues and establishes long-lived viral reservoirs. These reservoirs include the CNS, in which macrophage-lineage cells, and as suggested by many studies, astrocytes, may be infected. Here, we have investigated astrocyte infection by HIV-1. We confirm that astrocytes trap and internalize HIV-1 particles for subsequent release but find no evidence that these particles infect the cell. Astrocyte infection was not observed by cell-free or cell-to-cell routes using diverse approaches, including luciferase and GFP reporter viruses, fixed and live-cell fusion assays, multispectral flow cytometry, and super-resolution imaging. By contrast, we observed intimate interactions between HIV-1-infected macrophages and astrocytes leading to signals that might be mistaken for astrocyte infection using less stringent approaches. These results have implications for HIV-1 infection of the CNS, viral reservoir formation, and antiretroviral therapy.

INTRODUCTION

The CNS is a target of HIV-1 infection and serves as a viral reservoir that may not be readily accessed by antiretroviral drugs (Gray et al., 2016; Saylor et al., 2016). Approaches to HIV-1 eradication will therefore need to take this into account. Moreover, HIV-1 infection drives progressive cognitive impairment, incompletely prevented by antiretroviral therapy (ART), indicating ongoing pathological processes in the brain (Saylor et al., 2016; Spudich and González-Scarano, 2012). Perivascular macrophages and microglia are productively infected in the CNS and are considered to contribute to local inflammation and neuronal tissue degeneration (Brown, 2015; Rappaport and Volsky, 2015; Yadav and Collman, 2009).

Astrocytes are the most abundant brain cells and are the predominant neuro-glial cells involved in brain plasticity and neuroprotection (Churchill and Nath, 2013). Multiple studies, both in vitro and ex vivo, have implicated HIV-1 infection of astrocytes, although the nature of the infection is obscure. Ex vivo analyses of post-mortem brain tissue from HIV-1-infected individuals revealed viral proteins, viral nucleic acid, and virions associated with astrocytes (Churchill et al., 2009; Takahashi et al., 1996; Trillo-Pazos et al., 2003), and laser-capture experiments detected integrated viral DNA in nuclei isolated from astrocytes (Churchill et al., 2006). In vitro, rare primary astrocytes are reported as HIV-1 infected (Chauhan and Khandkar, 2015; Churchill and Nath, 2013; Nath, 2015), although this infection appears not to be permissive, because evidence is lacking for de novo infectious viral production (Chauhan and Khandkar, 2015; Churchill and Nath, 2013; Di Rienzo et al., 1998; Gorry et al., 1998, 2003; Hao and Lyman, 1999; Nath, 2015; Sabri et al., 1999; Vijaykumar et al., 2008). Lack of productive infection is consistent with failure to detect expression of the HIV-1 receptor CD4 on primary astrocytes and immortalized lines (Boutet et al., 2001; Ma et al., 1994; Peudenier et al., 1991; Sabri et al., 1999; Willey et al., 2003) and lack of consensus regarding CCR5 or CXCR4 expression (Rezaie et al., 2002; Sabri et al., 1999; Willey et al., 2003). Transfection of HIV-1 proviral DNA (Dewhurst et al., 1987a, 1987b; Shahabuddin et al., 1992) or vesicular stomatitis virus G protein (VSV-G)-pseudotyped HIV-1 transduction (Canki et al., 2001; Chauhan, 2015; Gray et al., 2013) into astrocytes revealed that bypassing the entry step allows HIV-1 to complete its life cycle and produce new viral RNA, proteins, and infectious particles, demonstrating the absence of major intracellular blocks to HIV-1 replication. Altogether, these data reveal a puzzling inconsistency: if HIV-1 can enter astrocytes using unconventional CD4-independent entry pathways and post-entry HIV-1 is replication competent, why does permissive viral replication not proceed in these cells?

Alternative explanations of astrocyte infection by HIV-1 have been proposed. First, it has been suggested that cell-to-cell transfer of HIV-1 from infected T cells to astrocytes may overcome entry barriers to cell-free infection, allowing astrocyte infection (Do et al., 2014; Luo and He, 2015; Nath et al., 1995), potentially via a CD4-independent but CXCR4-dependent mechanism (Li et al., 2015). Second, cell-free virus capture and

This is an open access article under the CC BY license (http://creativecommons.org/licenses/by/4.0/).

endocytosis by astrocytes have been observed (Chauhan and Khandkar, 2015; Chauhan et al., 2014; Clarke et al., 2006; Deiva et al., 2006; Gray et al., 2014; Hao and Lyman, 1999; Liu et al., 2004), establishing, at least in vitro, a transient reservoir of infectious virus within a CD81^{+} intracellular compartment (Gray et al., 2014) that can be released and transferred in trans to permissive cells. Although this model may explain detection of viral proteins, nucleic acids, and infectious virus in astrocytes, it does not explain apparently integrated viral genomes associated with astrocytes in ex vivo brain tissue from infected individuals or in vitro cocultures with infected permissive cell types.

An alternative explanation for detection of viral proteins, nucleic acids, and integration products associated with astrocytes may result from their phagocytic capacity. Astrocytes engulf damaged and dying cells or their fragments as a mechanism to avoid local necrosis and subsequent CNS inflammation (Chang et al., 2000; Lööv et al., 2012, 2015; Magnus et al., 2002; Sokolowski et al., 2011). Engulfed cells may be maintained for long periods with minimal degradation (Lööv et al., 2015), potentially explaining the observation of astrocytes with a multinuclear morphology (Lööv et al., 2012). We have demonstrated that macrophages avidly engulf HIV-1-infected and dying T cells, leading to macrophage infection in the case of macrophage-tropic viruses (Baxter et al., 2014). However, when T cells infected with non-macrophage-tropic HIV-1 were engulfed by macrophages, they became positive for viral antigens but were not productively infected (Baxter et al., 2014; Sattentau and Stevenson, 2016). We therefore hypothesized that astrocytes might, because of recognition of stressed, damaged, and dying cells, engulf HIV-1-infected cells and/or cell debris, thereby becoming positive for viral antigens and nucleic acids without becoming infected.

Here we test this hypothesis using advanced reporter and imaging techniques to probe primary human fetal astrocyte (HFA) cultures exposed to cell-free HIV-1 or infected primary macrophages, the most biologically relevant HIV-1 target cell in the brain. Despite observing uptake and release of HIV-1 particles with the potential to propagate limited infection in trans as observed by others (Chauhan and Khandkar, 2015; Chauhan et al., 2014; Clarke et al., 2006; Gray et al., 2014), we found no evidence for virus-mediated fusion with, or productive infection of, astrocytes after either cell-free or cell-to-cell spread. Coculture of astrocytes with infected macrophages led to tight contact between these cell types, with evidence of astrocyte internalization of virions and infected macrophages and macrophage-derived cellular material but no evidence of astrocyte infection. We therefore suggest astrocyte uptake of virus and material derived from infected cells as an explanation for the apparent infection of astrocytes reported by others, and we conclude that astrocytes are unlikely to function as long-term autonomous viral reservoirs within the CNS.

RESULTS

Astrocytes Are Not Productively Infected by Cell-free HIV-1

Although previous studies have suggested that cell-free HIV-1 may enter and infect astrocytes via unconventional and largely undefined entry pathways, most studies used wild-type HIV-1 with infection readout as release of cell-free viral p24 or infectious virions. Because this approach cannot differentiate between true infection and virion uptake followed by subsequent release, we used replication-competent luciferase reporter (LucR) viruses to probe HFA infectivity. LucR viruses give a sensitive readout of viral infection by reporting on viral entry, reverse transcription, and luciferase translation (Edmonds et al., 2010). We used primary HFAs as in multiple other HIV-1 infection studies, and we compared their permissivity to infection with monocyte-derived macrophages (MDMs) and highly permissive TZM-bl reporter cells (Montefiori, 2009). The CCR5-tropic (R5) viruses HIV-1BaL-LucR and HIV-1YU2-LucR and the CXCR4-using (X4) virus HIV-1NL4.3LucR were produced by transient 293T transfection and titrated on TZM-bl cells to obtain infectious titer and MOI. Viruses were added at an MOI of 0.1 (based on the TZM-bl cell infection) to similar numbers of TZM-bl cells, MDMs, or HFAs for 24 hr and washed, and the cells cultured for up to 10 days. At the time points shown, cells were lysed and assayed for luciferase signal and culture supernatant was analyzed for p24 content (Figure 1). Azidothymidine (AZT)-sensitive infection of TZM-bl cells by all viruses peaked at day 2 and waned with time as a result of HIV-1-induced syncytium formation and cell death, which was paralleled by a rapid and prolonged supernatant p24 signal. R5 virus infection kinetics were slower in MDMs than in TZM-bl cells but yielded increasing AZT-sensitive luciferase expression over time, with similar p24 release dynamics. Consistent with its non-macrophage tropism, HIV-1NL4.3-LucR virus failed to productively infect MDMs. By contrast with TZM-bl cells and MDMs, HFAs failed to produce luciferase activity significantly above background with any virus and at any time point (Figures 1E and 1G). Low-level supernatant p24, ~10- to 20-fold less than that measured in TZM-bl cells and MDMs, was detected in HFA supernatants with or without AZT treatment over 10 days (Figure 1H). Using sensitive luciferase reporter viruses, these results confirm previous findings that astrocytes pulsed with cell-free HIV-1 are not productively infected but rather adsorb and release viral p24 over an extended period. Previous studies have failed to detect CD4 and CCR5 on human astrocytes (Sabri et al., 1999; Willey et al., 2003), whereas CXCR4 expression has been implicated in astrocyte infection (Li et al., 2015). We investigated this by surface labeling with monoclonal antibodies (mAbs) against CD4, CCR5, and CXCR4 in live HFAs or for receptor expression within intracellular vesicular compartments in which HIV-1 might fuse by fixing and permeabilizing HFAs (Figure S1). Between 50% and 100% (Figure S1D) of live and fixed-permeabilized CD4+ Jurkat T cells and MDMs, gated on isotype control antibody, expressed moderate to high levels of CD4 and CCR5 quantified by the geometric mean fluorescence intensity (GMFI) (Figure S1E). By contrast, HFAs had no detectable signals above background under any condition. CXCR4 was expressed on Jurkat T cells, MDMs, and HFAs under all conditions, with a signal that increased after fixation and permeabilization, suggesting preferential reactivity of this antibody with fixed CXCR4 and/or CXCR4 expression both at the surface and in intracellular compartments.

OPEN ACCESS
CellPress

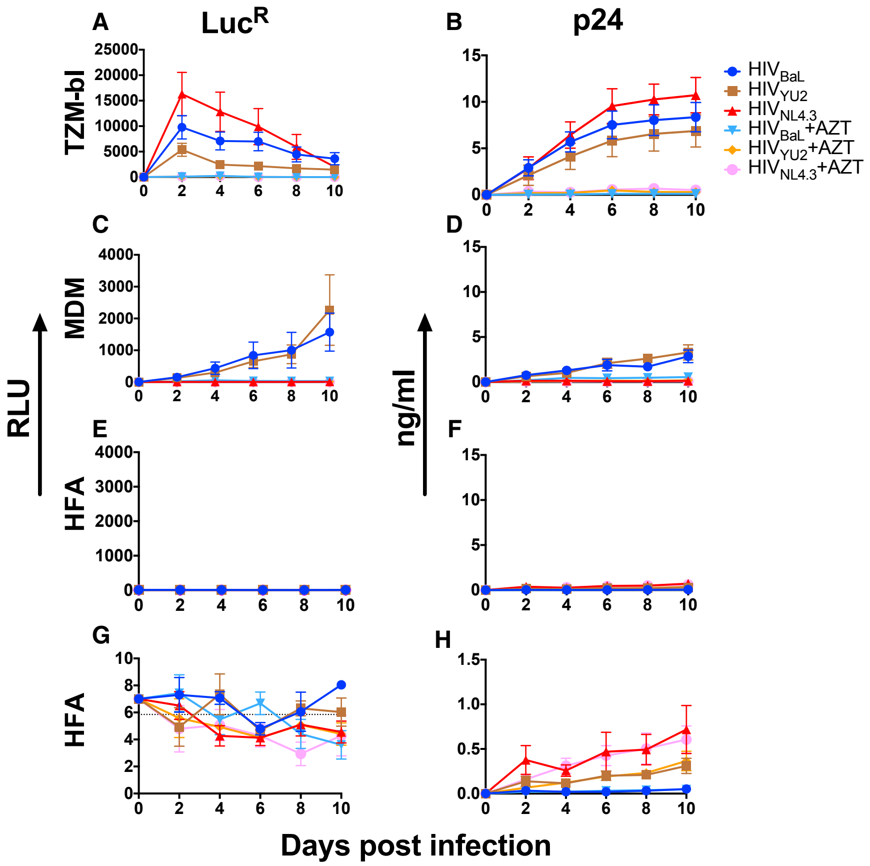

Figure 1. HFAs Are Not Productively Infected by HIV-1 but Trap and Release Viral Gag

(A–H) TZM-bl cells (A, B), MDMs (C, D), and HFAs (E–H) were incubated overnight with or without AZT with HIV-1 luciferase reporter viruses at MOI = 0.1, washed, and cultured with or without AZT. At the times shown, cells were lysed and assayed for luciferase activity (A, C, E, and G) or supernatants were harvested and assayed for Gag p24 by ELISA (B, D, F, and H). (G) and (H) represent (E) and (F), respectively, with expanded scales. The dotted line in (G) shows the background luciferase signal obtained with uninfected HFAs. Data represent means of three to five independent experiments ± SD using five MDM donors.

Cell-free HIV-1 Does Not Fuse with Astrocytes

Our data are consistent with infectious virion uptake into astrocytes without subsequent infection. To probe this in more detail, we carried out the well-characterized BlaM-Vpr assay, in which fusion of pseudoviruses (PVs) carrying a β-lactamase-Vpr fusion protein is reported by enzyme-catalyzed cleavage of the CCF2-AM substrate within the target cell cytoplasm, driving a green-to-blue change (Cavrois et al., 2002). Reporter PVs expressing BaL or LAI HIV-1 *env* or VSV-G were concentrated and spinoculated onto HFA or MDM target cells to generate a robust input signal and were live-cell gated and assessed for viability before fixation and flow cytometric analysis (Figures S2A–S2C). Figure 2B shows that uninfected MDMs expressed no significant signal, whereas the VSV-G PV yielded ~96% positive cells inhibited to ~38% by chloroquine, an antagonist of endosomal acidification (Figure 2C). HIV-1_{BaL} PV gave ~57% fusion with MDMs that was receptor-mediated, because the CD4 blocking mAb Q4120, the CCR5 antagonist TAK779 and the gp41 fusion inhibitor T20 reduced entry signals to background levels, whereas the CXCR4 antagonist AMD31000 failed to inhibit (Figure 2D). When HFAs were exposed to the same PV stocks, VSV-G PV transduced 99% of cells reduced to ~49% by chloroquine, whereas HIV-1_{BaL} gave close to background signals that were not further reduced by receptor antagonists or T20 (Figures 2G and 2H). PV carrying the X4 *env* LAI failed to produce a significantly inhibitable signal in MDMs or HFAs, confirming a lack of entry into either cell type (Figure 2H; Figure S2). Altogether, these results indicate that unlike VSV-G, HIV-1 Env is unable to mediate fusion with astrocytes. However, we did detect low-frequency (<–2%) β-lactamase signals in HFAs that were not significantly reduced by entry inhibitors (Figure 2H). We hypothesized that this apparently non-specific substrate conversion arose from the spinoculation and/or fixation process associated with the reported HIV-1 binding activity of astrocytes (Chauhan and Khandkar, 2015; Chauhan et al., 2014; Clarke et al., 2006; Deiva et al., 2006; Gray et al., 2014; Hao and Lyman, 1999; Liu et al., 2004). To exclude this, we used a modified BlaM-Vpr assay that generates real-time data in live cells without spinoculation or fixation (Putcharoen et al., 2012). BlaM-Vpr HIV-1 without Env (HIV-ΔEnv) or pseudotyped with the R5 HIV-1_{JRFL} Env or VSV-G were incubated with CCF2-AM substrate-loaded cells at 4°C for 30 min before washing, warming to 37°C, and imaging (Figure 3A). The ratio of uncleaved to cleaved substrate was quantified pixel by pixel and plotted against time, using the signal derived from HIV-1ΔEnv virions at the final time point as a background control for no fusion. Individual MDMs showed a positive BlaM fusion signal from 20 min onward when exposed to HIV_{JRFL} and HIV-$1_{VSV\text{-}G}$, with ~30% and ~16% red cells, respectively, by the final time point (Figures 3B–3D). By contrast, although HIV-$1_{VSV\text{-}G}$ yielded ~35% red cells at 140 min in HFAs, none were detectable at any time point with HIV-1_{JRFL} (Figures 3E–3G). Further evidence for an inability of HIV-1_{JRFL} to fuse with HFAs was obtained in real time using single-particle tracking. Gag-GFP HIV-1 virions double-labeled with the red fluorescent DiD membrane dye, resulting in yellow fluorescent particles, were incubated with cells for 30 min at 4°C, washed, and imaged every 8–12 s at 37°C for the times shown (Figure 4). Virion fusion leads to DiD diffusion into the limited endosomal membrane, turning

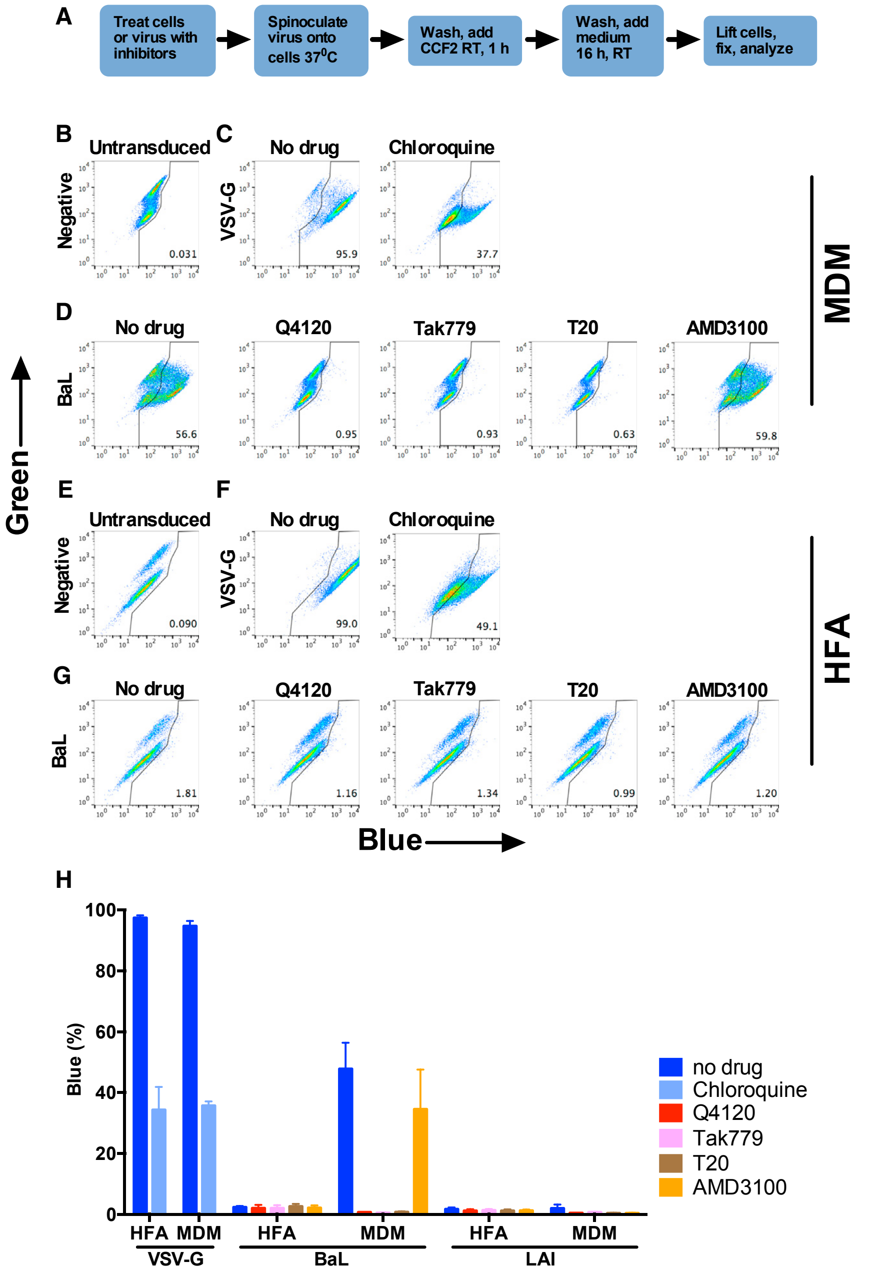

Figure 2. No Detection of HIV-1 Env Fusion with HFAs by BlaM-Vpr Assay

(A) Experimental plan.

(B–D) MDMs were untreated or treated with inhibitors and then mock-transduced (B) or transduced with VSV-G (C) or HIV-1 BaL Env-pseudotyped BlaM-Vpr HIV-1 (D) for 16 hr. Cells were then lifted, fixed, and analyzed by flow cytometry.

(E–G) HFAs were untreated or treated with inhibitors and then mock-transduced (E) or transduced with VSV-G (F) or HIV-1 BaL-pseudotyped BlaM-Vpr HIV-1 (G) for 16 hr. Cells were then lifted, fixed, and analyzed by flow cytometry.

(H) Means of data from three to five independent experiments ± SD using five MDM donors, including summarized results for LAI, presented in Figure S2.

it red, while the GFP-labeled capsid dissociates into the cytosol, leaving a red endosomal signal (Figure 4B) as previously described (Miyauchi et al., 2009). VSV-G fuses in an obligate pH-dependent manner from within endosomes, and HIV-1 Env fuses from within endosomes in MDMs, as previously demonstrated (Carter et al., 2011; van Wilgenburg et al., 2014). This is illustrated for HIV-$1_{VSV\text{-}G}$ fusion with HFAs in Figures 4D and 4E and Movie S1. A total of 28 fusion events (16%) for 250 HIV-$1_{VSV\text{-}G}$ tracked particles were detected in HFAs over 6 min in ten independent experiments (Figures 4F and 4G). By contrast, not a single fusion event was detected when 250 HIV-1_{JRFL} particles were tracked over the same time frame in HFAs (Figures 4G–4J; Movie S2). The slow loss of green fluorescence in Figure 4I represents a combination of particles moving out of focus and slow photobleaching of the green signal in the absence of fusion. However, both HIV-$1_{VSV\text{-}G}$ and HIV-1_{JRFL} particles fused readily with MDMs (Figure S3; Movies S3 and S4), confirming that this Env was fusion competent on permissive target cells. Altogether, these results confirm that HIV-1 particles cannot fuse with HFAs in live-cell systems over extended timescales during which VSV-G Env mediates multiple fusion events.

Transfer of HIV-1 from MDMs to HFAs by Cell-Cell Spread

Previous studies have reported that cell-cell spread of HIV-1 to astrocytes may overcome blocks imposed on cell-free virus infection of these cells, resulting in a low-level infection of astrocytes within the culture (Li et al., 2015; Luo and He, 2015; Nath et al., 1995). Alternatively, astrocytes may endocytose virions released from infected cells without becoming infected but leading to the appearance of infection. To interrogate these possibilities, we established an infected MDM-HFA coculture system (Figure 5A). We could not use a BlaM-Vpr assay to probe cell-to-cell spread, because we were unable to produce sufficiently high titer BlaM-Vpr PV in MDMs. We therefore infected MDMs with the R5 reporter virus HIV-$1_{JRFL\text{-}iGFP}$ for 7 days and cocultured them for 24 hr with HFAs. Cocultures were fixed and labeled for the astrocyte marker glial fibrillary acidic protein (GFAP) and imaged by confocal microscopy (CM). We observed a high percentage of HIV-1-infected MDMs associated with $GFAP^+$ HFAs, and the two cell types were confirmed to be interacting intimately making multiple intercellular contacts by scanning electron microscopy

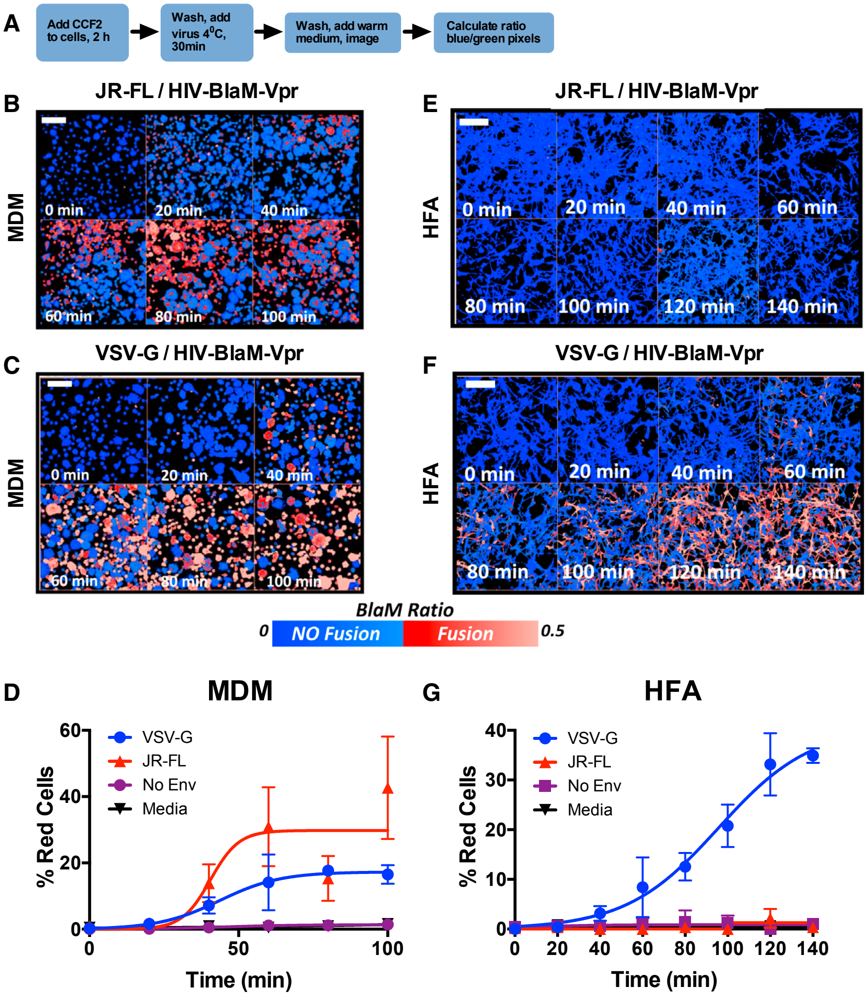

Figure 3. Live-Cell Fusion Assay Does Not Detect HIV-1 Env Fusion with HFAs

(A) Experimental plan.

(B and C) CCF2-loaded MDMs exposed to HIV-1 BlaM-Vpr pseudotyped with HIV-1 JRFL (B) or VSV-G Env (C) were imaged at the times shown.

(D) Quantified results from (B) and (C) showing the percentage of red cells as a function of time with the sigmoidal curve fit. Mean of three independent experiments ± SD using three MDM donors.

(E and F) CCF2-loaded HFAs exposed to HIV-1 BlaM-Vpr pseudotyped with HIV-1 JRFL (E) or VSV-G Env (F) were imaged at the times shown.

(G) Quantified results from (E) and (F), showing the percentage of red cells as a function of time with the sigmoidal curve fit. Mean of three independent experiments ± SD.

Blue pseudocolor represents no fusion; red represents fusion. Scale bar, 70 μm.

(Figures S4A and S4B). To probe the fate of individual HIV-1 particles transferred from MDMs to HFAs, we used super-resolution, stimulated emission depletion (STED) microscopy that has sufficient resolution to accurately image individual virions and viral Env (Chojnacki et al., 2012). HIV-1_{BaL}-infected MDMs were cocultured with HFAs for 3 or 24 hr, fixed, permeabilized, and labeled for GFAP and Gag p24 (Figures 5A and 5B). Fields containing GFAP$^+$ HFAs in close apposition to HIV-$1_{BaL}{}^+$ MDMs and in which virions were readily visible were selected for further analysis. At 3 hr post-coculture, virions were observed in proximity to, and associated with, HFA plasma membranes and frequently associated with astrocyte dendrites (Figure 5B) as previously described (Do et al., 2014). We quantified the association of virions with HFAs by acquiring *z* stacks of x-y images and counting individual virions within ~2 μm of the astrocyte membrane as identified by GFAP label (Figure 5A). Virion-plasma membrane distances were only measured in the x-y plane, because the resolution in the z plane was insufficient to quantify accurately, as exemplified in Figures 5C and 5E. Data from 203 individual virions are summarized in Figure 5F, showing that all virions were either proximal to or associated with the HFA plasma membrane, but none were beneath the plasma membrane, demonstrating a lack of virion internalization by 3 hr. By contrast, when infected MDMs were cocultured with HFAs for 24 hr (Figures 5D and 5E), most virions (192 counted) were beneath the plasma membrane, implying efficient virus internalization (Figure 5F). Virion internalization might result from fusion with the HFA membrane or from endocytic uptake as previously described (Chauhan and Khandkar, 2015; Chauhan et al., 2014; Clarke et al., 2006; Deiva et al., 2006; Gray et al., 2014; Hao and Lyman, 1999; Liu et al., 2004). To differentiate between these possibilities, we double-labeled virions for Gag and Env and compared the percentage of double-positive virions beneath the HFA membrane with that of the same virus sample bound to the solid phase. If virion entry into HFAs was fusion mediated, then the Env signal would dissociate from Gag and remain associated with the cell membrane, whereas endocytosis would leave the two signals associated on virions. Comparison of virions within HFAs after 24 hr coculture with infected MDMs (Figure 5G) to cell-free virions released from the same MDM culture and coated onto on a glass surface (Figure 5H) revealed 69.6% and 69.1%, respectively, with colocalized Gag and Env, demonstrating no loss of Env upon virion internalization into HFAs. These data, together with our other lack of evidence of virus-HFA fusion, strongly support the hypothesis that virions released from infected, contacting MDMs are endocytosed into HFAs without subsequent fusion.

HFAs Interact Intimately with and Engulf HIV-1-Infected MDM Material

HIV-1 proteins and nucleic acids, including integrated viral DNA, have been detected in astrocytes, yet we were unable to detect astrocyte infection in our systems. Although virions closely associated with HFA membranes would yield a signal positive for viral

OPEN
ACCESS
CellPress

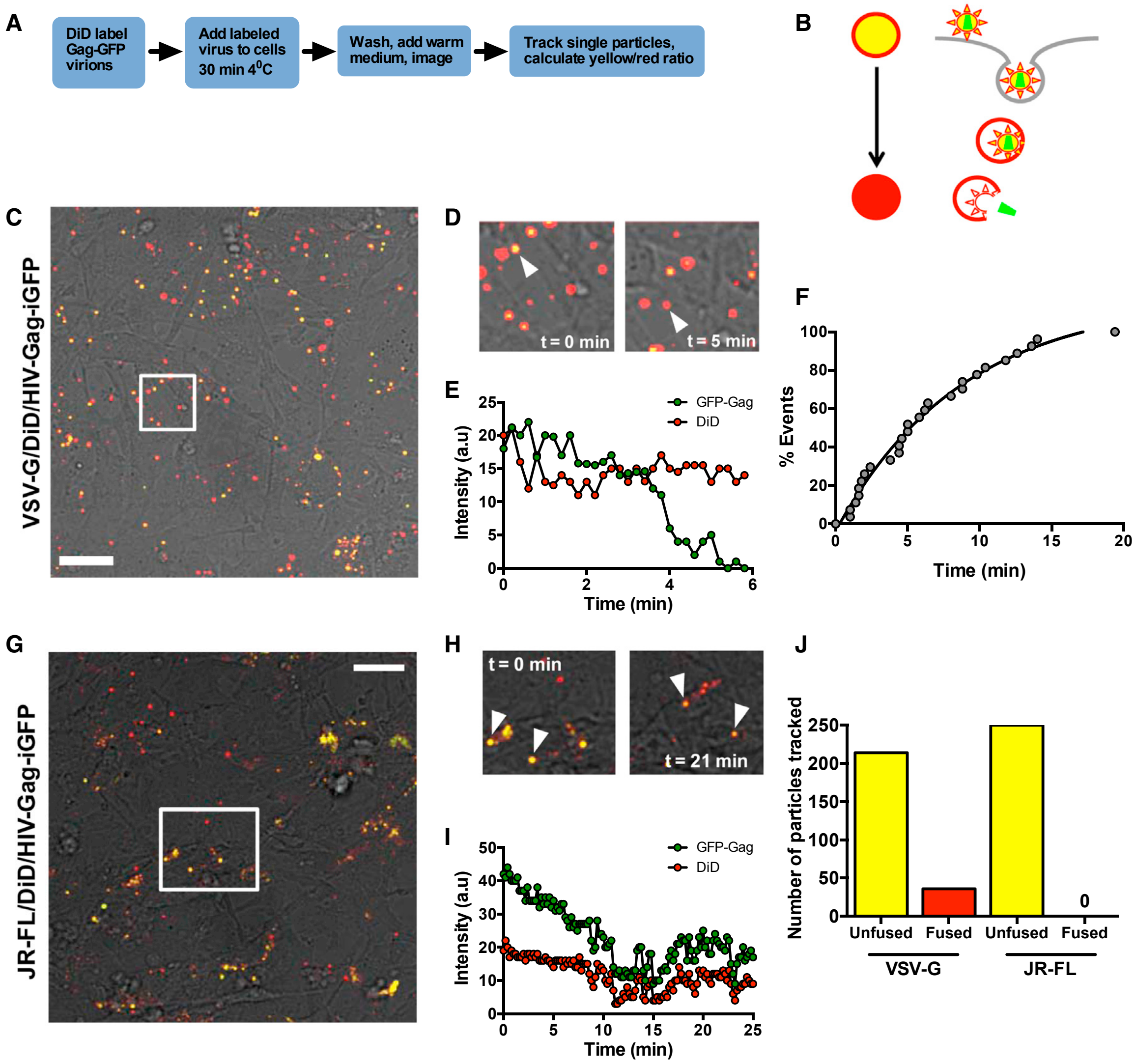

Figure 4. Single-Particle Tracking Fails to Reveal HIV-1 Env Fusion with HFAs

(A) Experimental plan.

(B) Cartoon of entry assay events. Yellow double-labeled (DiD, red; GFP, green) virions enter the cell by endocytosis. Fusion results in the red label entering the endosomal membrane and the green signal moving into the cytosol away from the plane of focus, leaving a red vesicular signal.

(C) Low-magnification x-y CM image of single- and double-labeled HIV-1-VSV-G particles on HFAs at T = 0. Arrowhead points to the double-labeled virion that fuses in (D). Scale bar, 10 μm.

(D) Close-up of white boxed area in (B) at T = 0 (left panel) and T = 5 min (right panel). Arrowheads point to the virion (yellow in the left panel) and the fusion event (red in the right panel). Region of interest represents 10 × 10 μm. See Movie S1.

(E) Data from the fusion event tracked in (C), in which the signal for the green channel is lost between 3.5 and 5 min while the red channel signal remains constant.

(F) Kinetic distribution of 28 single fusion events from four independent experiments.

(G) Low-magnification image of single- and double-labeled HIV-1-JRFL particles on HFAs at T = 0. Scale bar, 10 μm.

(H) Close-up of white boxed area in (G) at T = 0 (left panel) and T = 21 min (right panel). Arrowheads indicate double-labeled yellow virions. Region of interest is 15 × 12 μm. See Movie S2.

(I) Data quantified from a virion tracked in (H) in which the signal for red and green channels remains constant after initial photobleaching of the green signal between 0 and 10 min.

(J) Summary of fusion events totaling ~17% of all tracked yellow double-labeled particles (36 of 250) for VSV-G and 0% (0 of 250) for JR-FL. Data presented are from ten independent experiments using three MDM donors. See Figure S3 and Movies S3 and S4 for fusion data in MDMs.

proteins and nucleic acids, this would not explain the detection of viral nucleic acids from nuclei within astrocytes as previously described (Churchill et al., 2006). Based on the phagocytic activity of astrocytes (Chang et al., 2000; Lööv et al., 2012, 2015; Magnus et al., 2002; Sokolowski et al., 2011), we hypothesized that this discrepancy might be explained by astrocyte phagocytosis of infected MDMs and their debris eliciting false-positive infectivity signals. To probe this, we used multispectral

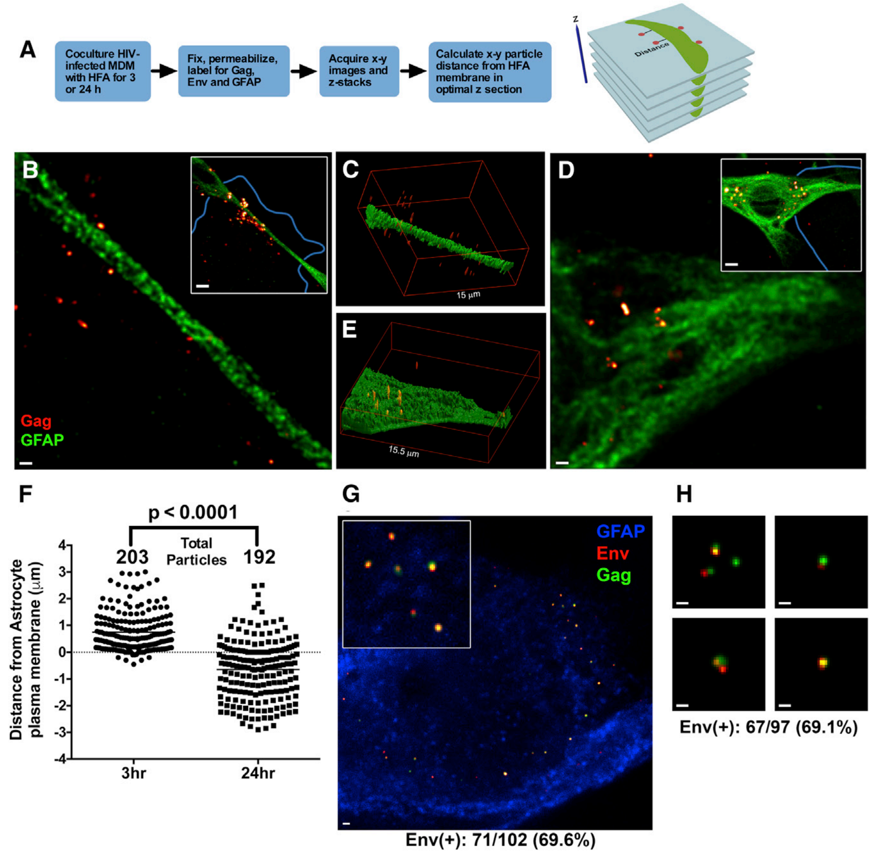

Figure 5. STED Analysis of HIV-1 Transfer from MDMs to HFAs

(A) Experimental chart and approach for measurement of virion-membrane distances.

(B) Coculture of HIV-1_{BaL}-infected MDMs with HFAs (GFAP, green) for 3 hr, with virions released from the MDMs labeled in red. Inset shows a low-magnification image, with MDMs outlined in blue. Scale bar, 500 nm; inset scale bar, 3 μm.

(C) 3D z projection of x-y sections through HFAs showing loss of resolution in the z plane.

(D) Coculture of HIV-1_{BaL}-infected MDMs with HFAs (GFAP, green) for 24 hr, with virions released from the MDMs in orange. Inset shows a low-magnification image, with MDMs outlined in blue. Scale bar, 500 nm; inset scale bar, 3 μm.

(E) 3D z projection of x-y sections through HFAs showing loss of resolution in the z plane.

(F) Distance of individual HIV-1 particles from HFA plasma membrane at 3 and 24 hr post-MDM-HFA coculture. Dotted line represents membrane, black line is mean, and $p < 0.0001$, determined by Student's t test. Data are from two independent experiments using two MDM donors.

(G) 3D z projection of Env/Gag-labeled HIV-1 particles associated with an HFA after 24 hr coculture with infected MDMs, of which >50% are internalized into HFAs. Scale bar, 500 nm; inset scale bar, 200 nm.

(H) Representative images of Gag/Env-labeled HIV-1 particles adhered to a coverslip. Scale bar, 200 nm.

flow cytometry (ImageStream), which allows phenotypic and quantitative analysis of cell-cell interactions as previously described for MDM uptake of HIV-1-infected T cells (Baxter et al., 2014). MDMs infected for 7 days with HIV-$1_{BaL\text{-}iGFP}$ were cocultured with DiD-labeled HFAs for 24 hr before lifting, fixation, permeabilization, labeling for CD14, and analysis (Figure 6A). Gating for single cells and multiple-cell conjugates was carried out as described (Figure S5), and images were acquired. Figures 6B–6D show example images of an infected single MDM ($CD14^+/GFP^+$), uninfected HFAs (DiD^+), and tight clusters of infected MDMs and HFAs. Quantification of these events (Figure 6G) revealed clustering of ~14% ± 6% of HFAs and MDMs, of which 2.8 ± 2% were HIV-1^+. Of particular significance, we observed combined "single" $CD14^+/DiD^+/GFP^-$ events representing MDMs engulfed by HFAs (or potentially HFAs engulfed by MDMs) (Figure 6E) and $CD14^+/DiD^+/GFP^+$ events representing infected MDMs engulfed by HFAs or possibly HFAs engulfed by infected MDMs (Figure 6F). Quantification of these single events revealed 10% ± 8% double positive for HFA and MDM markers and 1.6% ± 1.4% triple positive for HFAs, MDMs, and HIV-1. These images clearly reveal not only that MDMs and HFAs engage tightly into clusters but also that HFAs may engulf both uninfected MDMs, presumably damaged and dying cells, and infected MDMs, leading to apparent single cells triple positive for astrocyte, macrophage, and HIV-1 markers. Because the resolution of these images was insufficient to determine whether HFAs had engulfed MDM material, or vice versa, we carried out 3D confocal microscopy. HIV-1-infected MDMs were lifted and cocultured with HFAs for 24 hr and then fixed, permeabilized, and labeled for GFAP (HFAs) and HIV-1 Gag. Serial x-y optical sections were acquired in the z plane, and 3D reconstructions are shown in Figures 6H–6K and associated Movies S5 and S6. Figures 6H–6K show two independent HFAs that had engulfed infected MDM material, the dimensions of which (multiple ~1 μm infected MDM-derived vesicles in Figures 6H and 6I and a ~5 μm infected MDM-derived fragment in Figures 6J and 6K) represent infected cellular material. Analysis of infected MDMs cocultured with HFAs for 24 hr (as imaged by CM and scanning EM in Figures S4A and S4B, respectively) by transmission electron microscopy (TEM) revealed an infected MDM partially engulfed by an HFA (Figure S4C) and an apparently uninfected MDM potentially engulfed by an HFA (Figure S4D). Altogether, these results confirm that astrocytes can closely interact with and engulf infected macrophages and macrophage-derived material that would give the appearance, in low-resolution images and by assay of viral nucleic acids and proteins, of infected astrocytes.

DISCUSSION

Our results demonstrate unequivocally that cell-free HIV-1 is unable to fuse with and infect HFAs, in line with the absence of CD4

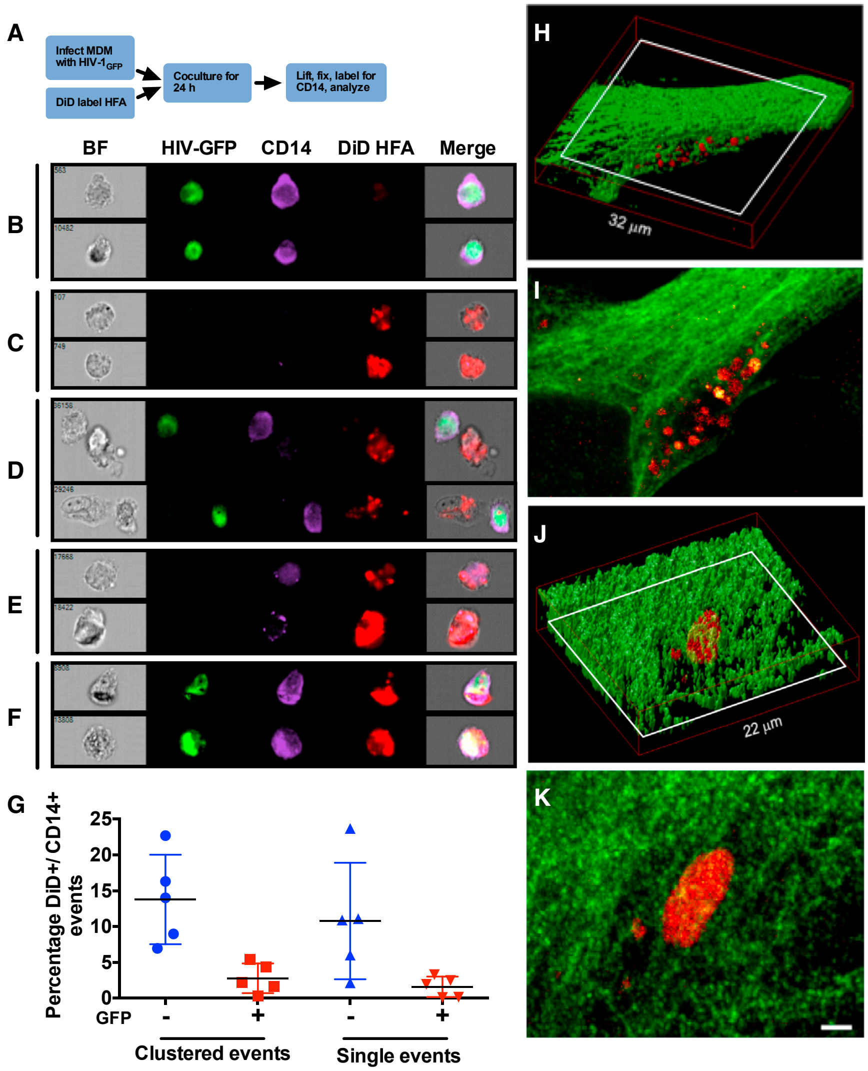

Figure 6. HFAs Interact with and Engulf HIV-1-Infected MDM Material

(A) Experimental plan.

(B) Single infected MDMs (two independent examples) showing bright-field (BF) morphology and DiD^{-}/GFP^{+}/CD14^{+} phenotype.

(C) Single HFAs (two independent examples) showing BF morphology and DiD^{+}/GFP^{-}/CD14^{-} phenotype.

(D) Clustered cells (two independent examples) showing BF morphology of conjugates of infected MDMs (DiD^{-}/GFP^{+}/CD14^{+}) and HFAs (DiD^{+}/GFP^{-}/CD14^{-}).

(E) Combined single events (two independent examples) of uninfected MDMs (DiD^{-}/GFP^{-}/CD14^{+}) and HFAs (DiD^{+}/GFP^{-}/CD14^{-}).

(F) Combined infected MDM (DiD^{-}/GFP^{+}/CD14^{+}) and HFA (DiD^{+}/GFP^{-}/CD14^{-}) single events (two independent examples).

(G) Quantification of clustered cells and single events showing individual datum points and means from data collected in five independent experiments, each with 5,000–10,000 events analyzed with four MDM donors ± SD. See Figure S5 for the ImageStream gating strategy.

(H) HIV-1-infected MDMs and HFAs were cocultured for 24 hr, immunostained for GFAP (green) and p24 (red), and imaged by CM. The z projection of optical slices corresponds to the astrocyte body; the red box indicates dimensions. See Movie S5.

(I) Single x-y optical section though the white boxed region of the HFAs in (H). Scale bar, 2 μm.

(J) HIV-1-infected MDMs and HFAs were cocultured, labeled, and imaged as for (H). The red box indicates dimensions. See Movie S6.

(K) Single x-y optical section though the white boxed region of the HFAs in (J). Scale bar, 2 μm. See also Figure S4 for TEM images of MDMs interacting with and engulfed by HFAs.

and CCR5 expression. However, astrocytes adsorb and internalize HIV-1 virions and engulf fragments of HIV-1-infected macrophages via "eat me" signals that are likely to derive from cell stress, damage, and death (Chang et al., 2000; Lööv et al., 2012, 2015; Magnus et al., 2002; Sokolowski et al., 2011), although we cannot exclude additional infection-specific recognition signals. We therefore propose that previous reports of HFA infection in many in vitro and ex vivo analyses may be explained as follows. First, astrocyte adsorption and uptake of cell-free virus particles would lead to detection of infectious virions, viral proteins, and nucleic acids in cultured or ex vivo analyzed astrocytes (An et al., 1999; Churchill et al., 2009; Takahashi et al., 1996; Trillo-Pazos et al., 2003). Second, astrocyte binding to, and engulfment of, HIV-1-infected permissive cells and subcellular material either in coculture (as we have seen for infected MDMs) or in vivo would likewise lead to detection of astrocyte-associated viral proteins and nucleic acids that could be mistaken for infection. Astrocyte engulfment of entire infected cells could lead to detection of integrated viral DNA within permissive cell nuclei inside astrocytes, as described ex vivo in HIV-1-infected brain tissue (Churchill et al., 2006) and be interpreted as astrocyte infection. Of particular relevance to this concept, Churchill et al. (2009) reported that detection of HIV-1 DNA in astrocytes correlated with proximity to perivascular macrophages, which are productively infected in vivo (Brown, 2015; Spudich and González-Scarano, 2012). Also relevant to this hypothesis, myeloid cells isolated from HIV-1-infected patients (Josefsson et al., 2013) and simian immunodeficiency virus (SIV)-infected macaques (Calantone et al., 2014) were positive both for HIV-1 DNA and for rearranged T cell receptor sequences, implying myeloid cell phagocytosis of HIV-1-infected T cells. By contrast with astrocytes, however, macrophages express CD4 and CCR5 and are therefore permissive for HIV-1 infection following infected T cell engulfment (Baxter et al., 2014; Sattentau and Stevenson, 2016).

Our data are consistent with a model proposed by others in which virions adhere to, and are taken up by, HFAs into a compartment from which small amounts of infectious virus

may subsequently be released (Chauhan and Khandkar, 2015; Chauhan et al., 2014; Clarke et al., 2006; Deiva et al., 2006; Gray et al., 2014; Hao and Lyman, 1999; Liu et al., 2004). Astrocytes with engulfed infected material from permissive cells may also transiently release virus, although we have not directly demonstrated this in the current study. However, in neither case would astrocytes be likely to form a long-lived viral reservoir, because virus release from such archives would depend entirely upon the production of new virus in the CNS by permissive cells such as microglia and perivascular macrophages.

We, like many others (Chauhan, 2015; Do et al., 2014; Gray et al., 2013, 2016; Nath et al., 1995), have used HFAs as a source of target cells in this study. Although primary cells, they are fetal and therefore are likely to differ phenotypically from adult astrocytes. Another potential source of adult astrocytes is post-operative neural tissue. However, these astrocyte preparations are not homogeneous, and even very small proportions of contaminating $CD4^+$ T cells or macrophage-lineage cells would confound interpretation of infectivity analyses. Appropriately differentiated stem cells might provide alternative sources of astrocytes, but at present differentiation results in heterogeneous cell populations whose biological relevance to tissue-derived astrocytes remains to be fully defined (Hill et al., 2016). We cannot exclude the possibility that astrocytes in situ exposed to the neural tissue environment may be induced to express CD4 and CCR5 and therefore, along with endogenous CXCR4 expression, be susceptible to HIV-1 infection in vivo. Our study nevertheless provides compelling evidence that primary human astrocytes are not infectible in vitro and suggests testable hypotheses regarding the nature of HIV-1 nucleic acid and protein signatures associated with these cells. Future studies should therefore focus on high-resolution imaging analyses of astrocytes in neural tissue to determine whether HIV-1 signals originate from engulfment of infected material rather than true infection.

EXPERIMENTAL PROCEDURES

Cells and Tissue Culture

Human fetal astrocytes (HFAs) (ScienCell Research Laboratories), isolated from second trimester fetal cerebral cortex, were cultured in astrocyte media (ScienCell Research Laboratories) with 1% penicillin and streptomycin (P/S), 2% fetal bovine serum (FBS), and 1% astrocyte growth supplement (optimized insulin, transferrin, fibroblast growth factor, insulin-like growth factor 1, hydrocortisone, and progesterone). Blood-derived monocytes were isolated from peripheral blood mononuclear cells using Human Monocyte Isolation Kit II (Miltenyi Biotec) according to manufacturer's instructions. Monocytes were cultured in X-vivo (Lonza) with 1% heterologous human serum (Sigma) for 7 to 10 days, during which time they differentiated into monocyte-derived macrophages (MDMs). TZM-bl cells (NIH AIDS Research and Reference Reagent Program) and 293T cells were grown in DMEM (Sigma) with 1% P/S and 10% FBS.

Viruses

Viruses were prepared by transient transfection of 293T cells and titrated on TZM-bl cells. Also see Supplemental Information.

Cell-free Infection

HFAs (1×10^4), MDMs (1×10^5), and TZM-bl cells (1×10^4) were seeded in 96-well plates and infected for 24 hr with HIV-$1_{BaL\text{-}LucR}$, HIV-$1_{YU2\text{-}LucR}$, or HIV-$1_{NL4.3\text{-}LucR}$ at a MOI of 0.1, determined by titration on TZM-bl cells. In AZT-containing samples, 10 μM AZT were added to the cells 1 hr before infection. At 24 hr post-infection, virus was washed off and cells were incubated in ±10 μM AZT for a further 24 hr and then at 48 hr intervals until 10 days post-infection. For harvesting, cells were incubated in 60 μL GloLysis buffer (Promega) for 2 min before freezing at −80°C. Lysates were thawed and 50 μL were combined with 50 μL Renilla-Glo Luciferase reagent (Promega). Plates were analyzed in a SpectraMax M5 (Molecular Devices) using SpectraMax Pro v.5 with an integration of 1,000 ms. Supernatant p24 was assayed using an in-house p24 ELISA as previously described (Baxter et al., 2014). Briefly, inactivated and detergent (Empigen)-treated cell-free supernatants were incubated in ELISA plate wells pre-coated with anti-p24 antibody (D7320, Aalto), followed by detection with biotinylated anti-p24 antibody (BC1071-BIOT, Aalto). The signal was developed using TMB Turbo One-Step Substrate (Pierce) and stopped with 0.5 M H_2SO_4, and optical density 450 (OD_{450}) was determined.

BlaM-Vpr Fusion Assay

Fusion was assayed using the GeneBLAzer kit (Life Technologies) as described (Cavrois et al., 2002). Drug concentrations were 500 nM Tak779 (CFAR), 1 μM AMD3100 (CFAR), 7.5 μg/mL T20 (NIH AIDS Reagents), 10 μg/mL Q4120, and 50 μg/mL chloroquine (Sigma).

Live-Cell Real-Time BlaM-Vpr Assay

Cells were plated at 3×10^5 (MDMs) or 1×10^5 (HFAs) cells/well in 8-well μ-Slides (Ibidi). On the day of assay, cells were loaded with CCF2-AM from the LiveBLAzer FRET-B/G Loading Kit (Life Technologies) and incubated at room temperature (RT) in the dark for 2 hr with 12.5 mM probenecid. CCF2-AM was removed, and cells were washed with PBS and cooled on ice before the addition of PV harboring BlaM-Vpr ($HIV_{No\ Env}$, $HIV_{VSV\text{-}G}$, and $HIV_{JR\text{-}FL}$), MOI = 3 in 100 μL. Cells were incubated at 4°C for 30 min with 12.5 mM probenecid. Cells were washed with PBS to remove unbound particles, fresh medium containing 12.5 mM probenecid was added, and cells were imaged on a Leica SP8XSMD confocal microscope at 37°C. Observations were recorded from 300–500 cells/field, with three fields/experiment. Also see Supplemental Information.

Live-Cell Single-Virus Tracking

Single-virus tracking in live MDMs and HFAs was performed as in Jones and Padilla-Parra (2015) and is described in more detail in Supplemental Information.

ImageStream

MDMs infected with HIV-$1_{BaL\text{-}iGFP}$ were lifted with 12 mM lidocaine/5 mM EDTA in PBS and added to DiD (Molecular Probes)-labeled HFAs seeded at 5×10^6 cells/dish. Cells were cocultured for 24 hr before lifting and fixation for 15 min in 4% formaldehyde in PBS. Cells were permeabilized in 0.1% saponin (Sigma) and 30% FCS for 30 min at RT before labeling for CD14 (CD14-eFluor450, eBioscience). Cells were analyzed on an ImageStream X Mark II (Amnis) with a 20× or 40× objective with extended depth of field (EDF) and data processed using Ideas v.6 (Amnis).

Confocal and STED Microscopy

Adherent cells (MDMs and HFAs) were washed and fixed in 4% paraformaldehyde (PFA) for 15 min, followed by washing in PBS and storage in 1% BSA/PBS. For confocal and stimulated emission depletion (STED) microscopy, 37G12 (anti-p24) and 2G12 (anti-gp120) antibodies (Polymun) were coupled to ATTO 490LS (Atto-Tec) or Abberior STAR 635P (Abberior) dyes via n-hydroxysuccinimide (NHS)-ester chemistry following manufacturer's instructions. GFAP was visualized using rabbit anti-GFAP Ab (Abcam) and anti-rabbit ATTO 490LS (Atto-Tec) or Alexa 488 (Life Technologies). Also see Supplemental Information.

ACCESSION NUMBERS

The accession numbers for the flow cytometry data related to the fusion assay reported in this paper are FlowRepository: FR-FCM-ZYZM and FR-FCM-ZYZP.

SUPPLEMENTAL INFORMATION

Supplemental Information includes Supplemental Experimental Procedures, five figures, and six movies and can be found with this article online at http://dx.doi.org/10.1016/j.celrep.2017.01.027.

AUTHOR CONTRIBUTIONS

Q.J.S., T.D., J.C., and C.E. conceived the study; R.A.R., J.C., D.M.J., T.D., E.J., and S.P.-P. carried out the experiments; and all authors analyzed and interpreted the data. Q.J.S. and R.A.R. wrote the manuscript, and all authors edited the manuscript.

ACKNOWLEDGMENTS

J.C. and C.E. were supported by The Medical Research Council (grant MC_UU_12010 and unit programs G0902418 and MC_UU_12025), the Wolfson Foundation (grant 18272), MRC/BBSRC/EPSRC (grant MR/K01577X/1), Wellcome Trust (grant 104924/14/Z/14), and the Deutsche Forschungsgemeinschaft (grant 1905, Research unit 1905, "Structure and Function of the Peroxisomal Translocon"). The S.P.-P. research group is funded by the Nuffield Department of Medicine Leadership Fellowship from Oxford University. The Wellcome Trust Centre for Human Genetics is supported by Wellcome Trust (grant 090532/Z/09/Z). We thank the Cellular Imaging Core from the Wellcome Trust Centre for Human Genetics and the Wolfson Imaging Centre at the Weatherall Institute of Molecular Medicine for their support and input. Q.J.S. is a Jenner investigator and a James Martin senior fellow. Electron microscopy was performed at the Dunn School EM Facility, and we are grateful to Anna Pielach for preparing the EM samples.

Received: June 7, 2016
Revised: November 13, 2016
Accepted: January 12, 2017
Published: February 7, 2017

REFERENCES

An, S.F., Groves, M., Giometto, B., Beckett, A.A., and Scaravilli, F. (1999). Detection and localisation of HIV-1 DNA and RNA in fixed adult AIDS brain by polymerase chain reaction/in situ hybridisation technique. Acta Neuropathol. *98*, 481–487.

Baxter, A.E., Russell, R.A., Duncan, C.J., Moore, M.D., Willberg, C.B., Pablos, J.L., Finzi, A., Kaufmann, D.E., Ochsenbauer, C., Kappes, J.C., et al. (2014). Macrophage infection via selective capture of HIV-1-infected CD4+ T cells. Cell Host Microbe *16*, 711–721.

Boutet, A., Salim, H., Taoufik, Y., Lledo, P.M., Vincent, J.D., Delfraissy, J.F., and Tardieu, M. (2001). Isolated human astrocytes are not susceptible to infection by M- and T-tropic HIV-1 strains despite functional expression of the chemokine receptors CCR5 and CXCR4. Glia *34*, 165–177.

Brown, A. (2015). Understanding the MIND phenotype: macrophage/microglia inflammation in neurocognitive disorders related to human immunodeficiency virus infection. Clin. Transl. Med. *4*, 7.

Calantone, N., Wu, F., Klase, Z., Deleage, C., Perkins, M., Matsuda, K., Thompson, E.A., Ortiz, A.M., Vinton, C.L., Ourmanov, I., et al. (2014). Tissue myeloid cells in SIV-infected primates acquire viral DNA through phagocytosis of infected T cells. Immunity *41*, 493–502.

Canki, M., Thai, J.N., Chao, W., Ghorpade, A., Potash, M.J., and Volsky, D.J. (2001). Highly productive infection with pseudotyped human immunodeficiency virus type 1 (HIV-1) indicates no intracellular restrictions to HIV-1 replication in primary human astrocytes. J. Virol. *75*, 7925–7933.

Carter, G.C., Bernstone, L., Baskaran, D., and James, W. (2011). HIV-1 infects macrophages by exploiting an endocytic route dependent on dynamin, Rac1 and Pak1. Virology *409*, 234–250.

Cavrois, M., De Noronha, C., and Greene, W.C. (2002). A sensitive and specific enzyme-based assay detecting HIV-1 virion fusion in primary T lymphocytes. Nat. Biotechnol. *20*, 1151–1154.

Chang, G.H., Barbaro, N.M., and Pieper, R.O. (2000). Phosphatidylserine-dependent phagocytosis of apoptotic glioma cells by normal human microglia, astrocytes, and glioma cells. Neuro-oncol. *2*, 174–183.

Chauhan, A. (2015). Enigma of HIV-1 latent infection in astrocytes: an in-vitro study using protein kinase C agonist as a latency reversing agent. Microbes Infect. *17*, 651–659.

Chauhan, A., and Khandkar, M. (2015). Endocytosis of human immunodeficiency virus 1 (HIV-1) in astrocytes: a fiery path to its destination. Microb. Pathog. *78*, 1–6.

Chauhan, A., Mehla, R., Vijayakumar, T.S., and Handy, I. (2014). Endocytosis-mediated HIV-1 entry and its significance in the elusive behavior of the virus in astrocytes. Virology *456–457*, 1–19.

Chojnacki, J., Staudt, T., Glass, B., Bingen, P., Engelhardt, J., Anders, M., Schneider, J., Müller, B., Hell, S.W., and Kräusslich, H.G. (2012). Maturation-dependent HIV-1 surface protein redistribution revealed by fluorescence nanoscopy. Science *338*, 524–528.

Churchill, M., and Nath, A. (2013). Where does HIV hide? A focus on the central nervous system. Curr. Opin. HIV AIDS *8*, 165–169.

Churchill, M.J., Gorry, P.R., Cowley, D., Lal, L., Sonza, S., Purcell, D.F., Thompson, K.A., Gabuzda, D., McArthur, J.C., Pardo, C.A., and Wesselingh, S.L. (2006). Use of laser capture microdissection to detect integrated HIV-1 DNA in macrophages and astrocytes from autopsy brain tissues. J. Neurovirol. *12*, 146–152.

Churchill, M.J., Wesselingh, S.L., Cowley, D., Pardo, C.A., McArthur, J.C., Brew, B.J., and Gorry, P.R. (2009). Extensive astrocyte infection is prominent in human immunodeficiency virus-associated dementia. Ann. Neurol. *66*, 253–258.

Clarke, J.N., Lake, J.A., Burrell, C.J., Wesselingh, S.L., Gorry, P.R., and Li, P. (2006). Novel pathway of human immunodeficiency virus type 1 uptake and release in astrocytes. Virology *348*, 141–155.

Deiva, K., Khiati, A., Hery, C., Salim, H., Leclerc, P., Horellou, P., and Tardieu, M. (2006). CCR5-, DC-SIGN-dependent endocytosis and delayed reverse transcription after human immunodeficiency virus type 1 infection in human astrocytes. AIDS Res. Hum. Retroviruses *22*, 1152–1161.

Dewhurst, S., Bresser, J., Stevenson, M., Sakai, K., Evinger-Hodges, M.J., and Volsky, D.J. (1987a). Susceptibility of human glial cells to infection with human immunodeficiency virus (HIV). FEBS Lett. *213*, 138–143.

Dewhurst, S., Sakai, K., Bresser, J., Stevenson, M., Evinger-Hodges, M.J., and Volsky, D.J. (1987b). Persistent productive infection of human glial cells by human immunodeficiency virus (HIV) and by infectious molecular clones of HIV. J. Virol. *61*, 3774–3782.

Di Rienzo, A.M., Aloisi, F., Santarcangelo, A.C., Palladino, C., Olivetta, E., Genovese, D., Verani, P., and Levi, G. (1998). Virological and molecular parameters of HIV-1 infection of human embryonic astrocytes. Arch. Virol. *143*, 1599–1615.

Do, T., Murphy, G., Earl, L.A., Del Prete, G.Q., Grandinetti, G., Li, G.H., Estes, J.D., Rao, P., Trubey, C.M., Thomas, J., et al. (2014). Three-dimensional imaging of HIV-1 virological synapses reveals membrane architectures involved in virus transmission. J. Virol. *88*, 10327–10339.

Edmonds, T.G., Ding, H., Yuan, X., Wei, Q., Smith, K.S., Conway, J.A., Wieczorek, L., Brown, B., Polonis, V., West, J.T., et al. (2010). Replication competent molecular clones of HIV-1 expressing Renilla luciferase facilitate the analysis of antibody inhibition in PBMC. Virology *408*, 1–13.

Gorry, P., Purcell, D., Howard, J., and McPhee, D. (1998). Restricted HIV-1 infection of human astrocytes: potential role of nef in the regulation of virus replication. J. Neurovirol. *4*, 377–386.

Gorry, P.R., Ong, C., Thorpe, J., Bannwarth, S., Thompson, K.A., Gatignol, A., Vesselingh, S.L., and Purcell, D.F. (2003). Astrocyte infection by HIV-1: mechanisms of restricted virus replication, and role in the pathogenesis of HIV-1-associated dementia. Curr. HIV Res. *1*, 463–473.

Gray, L.R., Tachedjian, G., Ellett, A.M., Roche, M.J., Cheng, W.J., Guillemin, G.J., Brew, B.J., Turville, S.G., Wesselingh, S.L., Gorry, P.R., and Churchill, M.J. (2013). The NRTIs lamivudine, stavudine and zidovudine have reduced HIV-1 inhibitory activity in astrocytes. PLoS ONE *8*, e62196.

Gray, L.R., Turville, S.G., Hitchen, T.L., Cheng, W.J., Ellett, A.M., Salimi, H., Roche, M.J., Wesselingh, S.L., Gorry, P.R., and Churchill, M.J. (2014). HIV-1 entry and trans-infection of astrocytes involves CD81 vesicles. PLoS ONE *9*, e90620.

Gray, L.R., Brew, B.J., and Churchill, M.J. (2016). Strategies to target HIV-1 in the central nervous system. Curr. Opin. HIV AIDS *11*, 371–375.

Hao, H.N., and Lyman, W.D. (1999). HIV infection of fetal human astrocytes: the potential role of a receptor-mediated endocytic pathway. Brain Res. *823*, 24–32.

Hill, E., Nagel, D., Parri, R., and Coleman, M. (2016). Stem cell-derived astrocytes: are they physiologically credible? J. Physiol. *594*, 6595–6606.

Jones, D.M., and Padilla-Parra, S. (2015). Imaging real-time HIV-1 virion fusion with FRET-based biosensors. Sci. Rep. *5*, 13449.

Josefsson, L., von Stockenstrom, S., Faria, N.R., Sinclair, E., Bacchetti, P., Killian, M., Epling, L., Tan, A., Ho, T., Lemey, P., et al. (2013). The HIV-1 reservoir in eight patients on long-term suppressive antiretroviral therapy is stable with few genetic changes over time. Proc. Natl. Acad. Sci. USA *110*, E4987–E4996.

Li, G.H., Anderson, C., Jaeger, L., Do, T., Major, E.O., and Nath, A. (2015). Cell-to-cell contact facilitates HIV transmission from lymphocytes to astrocytes via CXCR4. AIDS *29*, 755–766.

Liu, Y., Liu, H., Kim, B.O., Gattone, V.H., Li, J., Nath, A., Blum, J., and He, J.J. (2004). CD4-independent infection of astrocytes by human immunodeficiency virus type 1: requirement for the human mannose receptor. J. Virol. *78*, 4120–4133.

Lööv, C., Hillered, L., Ebendal, T., and Erlandsson, A. (2012). Engulfing astrocytes protect neurons from contact-induced apoptosis following injury. PLoS ONE *7*, e33090.

Lööv, C., Mitchell, C.H., Simonsson, M., and Erlandsson, A. (2015). Slow degradation in phagocytic astrocytes can be enhanced by lysosomal acidification. Glia *63*, 1997–2009.

Luo, X., and He, J.J. (2015). Cell-cell contact viral transfer contributes to HIV infection and persistence in astrocytes. J. Neurovirol. *21*, 66–80.

Ma, M., Geiger, J.D., and Nath, A. (1994). Characterization of a novel binding site for the human immunodeficiency virus type 1 envelope protein gp120 on human fetal astrocytes. J. Virol. *68*, 6824–6828.

Magnus, T., Chan, A., Linker, R.A., Toyka, K.V., and Gold, R. (2002). Astrocytes are less efficient in the removal of apoptotic lymphocytes than microglia cells: implications for the role of glial cells in the inflamed central nervous system. J. Neuropathol. Exp. Neurol. *61*, 760–766.

Miyauchi, K., Kim, Y., Latinovic, O., Morozov, V., and Melikyan, G.B. (2009). HIV enters cells via endocytosis and dynamin-dependent fusion with endosomes. Cell *137*, 433–444.

Montefiori, D.C. (2009). Measuring HIV neutralization in a luciferase reporter gene assay. Methods Mol. Biol. *485*, 395–405.

Nath, A. (2015). Eradication of human immunodeficiency virus from brain reservoirs. J. Neurovirol. *21*, 227–234.

Nath, A., Hartloper, V., Furer, M., and Fowke, K.R. (1995). Infection of human fetal astrocytes with HIV-1: viral tropism and the role of cell to cell contact in viral transmission. J. Neuropathol. Exp. Neurol. *54*, 320–330.

Peudenier, S., Héry, C., Ng, K.H., and Tardieu, M. (1991). HIV receptors within the brain: a study of CD4 and MHC-II on human neurons, astrocytes and microglial cells. Res. Virol. *142*, 145–149.

Putcharoen, O., Lee, S.H., Henrich, T.J., Hu, Z., Vanichanan, J., Coakley, E., Greaves, W., Gulick, R.M., Kuritzkes, D.R., and Tsibris, A.M. (2012). HIV-1 clinical isolates resistant to CCR5 antagonists exhibit delayed entry kinetics that are corrected in the presence of drug. J. Virol. *86*, 1119–1128.

Rappaport, J., and Volsky, D.J. (2015). Role of the macrophage in HIV-associated neurocognitive disorders and other comorbidities in patients on effective antiretroviral treatment. J. Neurovirol. *21*, 235–241.

Rezaie, P., Trillo-Pazos, G., Everall, I.P., and Male, D.K. (2002). Expression of beta-chemokines and chemokine receptors in human fetal astrocyte and microglial co-cultures: potential role of chemokines in the developing CNS. Glia *37*, 64–75.

Sabri, F., Tresoldi, E., Di Stefano, M., Polo, S., Monaco, M.C., Verani, A., Fiore, J.R., Lusso, P., Major, E., Chiodi, F., and Scarlatti, G. (1999). Nonproductive human immunodeficiency virus type 1 infection of human fetal astrocytes: independence from CD4 and major chemokine receptors. Virology *264*, 370–384.

Sattentau, Q.J., and Stevenson, M. (2016). Macrophages and HIV-1: an unhealthy constellation. Cell Host Microbe *19*, 304–310.

Saylor, D., Dickens, A.M., Sacktor, N., Haughey, N., Slusher, B., Pletnikov, M., Mankowski, J.L., Brown, A., Volsky, D.J., and McArthur, J.C. (2016). HIV-associated neurocognitive disorder—pathogenesis and prospects for treatment. Nat. Rev. Neurol. *12*, 234–248.

Shahabuddin, M., Volsky, B., Kim, H., Sakai, K., and Volsky, D.J. (1992). Regulated expression of human immunodeficiency virus type 1 in human glial cells: induction of dormant virus. Pathobiology *60*, 195–205.

Sokolowski, J.D., Nobles, S.L., Heffron, D.S., Park, D., Ravichandran, K.S., and Mandell, J.W. (2011). Brain-specific angiogenesis inhibitor-1 expression in astrocytes and neurons: implications for its dual function as an apoptotic engulfment receptor. Brain Behav. Immun. *25*, 915–921.

Spudich, S., and González-Scarano, F. (2012). HIV-1-related central nervous system disease: current issues in pathogenesis, diagnosis, and treatment. Cold Spring Harb. Perspect. Med. *2*, a007120.

Takahashi, K., Wesselingh, S.L., Griffin, D.E., McArthur, J.C., Johnson, R.T., and Glass, J.D. (1996). Localization of HIV-1 in human brain using polymerase chain reaction/in situ hybridization and immunocytochemistry. Ann. Neurol. *39*, 705–711.

Trillo-Pazos, G., Diamanturos, A., Rislove, L., Menza, T., Chao, W., Belem, P., Sadiq, S., Morgello, S., Sharer, L., and Volsky, D.J. (2003). Detection of HIV-1 DNA in microglia/macrophages, astrocytes and neurons isolated from brain tissue with HIV-1 encephalitis by laser capture microdissection. Brain Pathol. *13*, 144–154.

van Wilgenburg, B., Moore, M.D., James, W.S., and Cowley, S.A. (2014). The productive entry pathway of HIV-1 in macrophages is dependent on endocytosis through lipid rafts containing CD4. PLoS ONE *9*, e86071.

Vijaykumar, T.S., Nath, A., and Chauhan, A. (2008). Chloroquine mediated molecular tuning of astrocytes for enhanced permissiveness to HIV infection. Virology *381*, 1–5.

Willey, S.J., Reeves, J.D., Hudson, R., Miyake, K., Dejucq, N., Schols, D., De Clercq, E., Bell, J., McKnight, A., and Clapham, P.R. (2003). Identification of a subset of human immunodeficiency virus type 1 (HIV-1), HIV-2, and simian immunodeficiency virus strains able to exploit an alternative coreceptor on untransformed human brain and lymphoid cells. J. Virol. *77*, 6138–6152.

Yadav, A., and Collman, R.G. (2009). CNS inflammation and macrophage/microglial biology associated with HIV-1 infection. J. Neuroimmune Pharmacol. *4*, 430–447.

Cell Reports, Volume *18*

Supplemental Information

Astrocytes Resist HIV-1 Fusion but Engulf Infected Macrophage Material

Rebecca A. Russell, Jakub Chojnacki, Daniel M. Jones, Errin Johnson, Thao Do, Christian Eggeling, Sergi Padilla-Parra, and Quentin J. Sattentau

Supplementary materials for Russell et al "Astrocytes resist HIV-1 fusion but engulf infected macrophage material"

Supplementary methods

Viruses

Replication-competent infectious molecular clones stably expressing either Renilla luciferase or GFP in an isogenic NL4.3 backbone (NL-LucR.T2A) encoding HIV-1 BaL, HIV-1 YU2 and HIV-1 NL4.3 *env* ectodomains in *cis* (Ochsenbauer et al., 2012) were prepared by transfection using polyethyleneimine (PEI, Sigma-Aldrich) in HEK-293T cells and titered on TZM-bl cells. Single cycle pseudovirus vectors were prepared by transfecting 293T cells with the vector backbone pNL4.3.Luc.R-E- (NIH AIDS Research and Reference Reagent Program) (He et al 1995), pMM310 (NIH AIDS Research and Reference Reagent Program) (Tobiume et al, 2003) expressing the Vpr-β-lactamase fusion protein and one of the following Env-expressing plasmids; pHEF-VSV-G (NIH AIDS Research and Reference Reagent Program)(Chang L-J, et al. 1999), BaL, JRFL and LAI (NIH AIDS Research and Reference Reagent Program). Pseudovirus vectors were titered on TZM-bl cells and titer determined using ID-50 software version 5.0. For the live cell BlaM assay, cells were transfected with 2µg pR8ΔEnv, 2µg Vpr-BlaM, 1µg pcREV and 3µg of the appropriate viral envelope (either VSV-G or the CCR5-tropic HIV-1 strain JR-FL) using GeneJuice (Novagen). At 12 hr post-transfection the transfection mixture-containing medium was removed, cells washed with PBS and fresh $DMEM_{comp}$ (lacking phenol red) added and cells incubated for a further 24 hr. At 48 hr post-transfection viral supernatants were harvested, aliquoted and stored at -80˚C. For single virus tracking, cells were transfected in the same manner with the following modification: at 12 hr post-transfection cells were washed with PBS then incubated with 10ml Optimem (Life Technologies) containing 10µM DiD (Life Technologies) for 4 hr. The staining mixture was removed, cells washed twice with PBS and fresh $DMEM_{comp}$ (lacking phenol red) added, and cells incubated for a further 24 hr prior to harvesting.

Flow cytometry

Cells were prepared for surface labeling flow cytometry using fluorochrome-conjugated monoclonal antibody (mAb) labeling and matching isotype controls. MAbs used in experiments were: CD4-APC (clone RPA-T4 Becton Dickinson, BD); CXCR4-APC (clone 12G5 Biolegend); CCR5 (clone HEK/1/85a Biolegend). Cells

were pelleted in a 96 well round bottom plate and incubated in PBS with 2% FBS/0.01% sodium azide (wash buffer, WB), 10 µg/mL normal human IgG and conjugated antibody for 1 hr at 4^0C. Cells were washed with in WB and fixed with 4% formaldehyde in PBS at RT for 15 mins. Cells were washed 3 times with PBS and re-suspended in PBS for analysis. For labeling of fixed cells, cells were fixed in 4% formaldehyde in PBS prior to staining, as described above. For labeling intracellular markers cells were fixed as above and permeabilized in WB containing 0.1% saponin prior to staining, as described above. Data from 5-10 x 10^3 events per sample were acquired using a FACS Calibur (BD). Analysis was with FlowJo V10 software.

BlaM-Vpr fusion assay

HFA and MDM, seeded in 12-well plates at $1x10^6$ and $7.5x10^4$ cells/well respectively, were spinoculated at 1200 x g at 37°C for 45 min with 800 IU of BlaM-Vpr-containing PV expressing BaL, LAI or VSV-G Env. Supernatant was removed and replaced with CCF2 prepared in CO_2-independent medium (CO_2-IM) (Life technologies) and cells incubated for 1 hr at room temerapture (RT) in the dark. Cells were washed 3 times in CO_2-IM and incubated for 16 hr at RT in the dark in CO_2-IM / 10% FBS / 2.5 mM probenecid (Sigma). Cells were lifted using trypsin (Life technologies), fixed and analyzed on a CyAn ADP (Dako). In drug treatment experiments, cells (or virus for T20), were pretreated for 1 hr at 37°C (or RT for T20), prior to spinoculation. Drug supernatant concentration was maintained at every stage of the assay.

Real time BLaM-Vpr assay image acquisition and analysis

Cells loaded with CCF2 were excited using a 405 nm continuous laser (Leica, Manheim) and the emission spectra between 430-560 nm recorded pixel by pixel (512 X 512) with a lambda resolution of 12 nm. The ratio of blue emission (440 - 480 nm, cleaved CCF2) to green (500 - 540 nm, un-cleaved CCF2) was calculated pixel by pixel using ImageJ (http://imagej.nih.gov/ij/) for three different 20 X objective observation fields in each well and plotted as a function of time. A blue/green threshold (fusion threshold) was set using Env-negative ($HIV_{No\ Env}$) virions containing BlaM-Vpr as a background control to provide a fusion detection limit that corresponded to 0.3+/- 0.05 BlaM ratio. The fusion threshold was calculated by recovering the signal (blue/green intensity ratio) coming from individual cells at the last time point (~100 min for MDM and ~140 min for HFA) plus 2 x SD from ~300 cells in the observation field. This threshold was then applied to all images at all time points of cells treated with $HIV_{No\ Env}$, HIV_{VSV-G} and HIV_{JR-FL}. Cells above the threshold

where pseudocolored in red while cells below the threshold where pseudocolored in blue. "Red" cells were then counted as a function of time to recover the number of fusogenic cells that corresponds to an accurate measure of viral fusion kinetics.

Live cell single particle image acquisition and analysis

Cells were plated at $3x10^5$ (MDM) or $1x10^5$ (HFA) cells/well in 8-well µ-Slides (Ibidi) and double-labelled virions (Gag-iGFP and DiD) were incubated for 30 min at 4 °C and cells washed with PBS to remove unbound virions. Cells were mounted onto the confocal Leica SP8XSMD with a stage maintained at 37 °C. Once a suitable image field was chosen, virus internalization was initiated by adding 1 mL of warm HBSS and imaging commenced using a Leica SP8 confocal microscope with a 63×/1.4 NA oil immersion objective and 2 HyD internal detectors capable of single photon counting. Slow loss of red and green signals over time reflects particle drift outside the plane of focus and some photobleaching in the green channel. True fusion events are represented by strict criteria (Jones and Padilla-Parra, 2015; Padilla-Parra et al., 2013; Padilla-Parra et al., 2014) defined by a rapid (~1 min) drop in either the green signal whilst the red color (DiD) remains stable (endosomal fusion). Images were acquired every 8–12 s for ~45 min. The axial position of a specimen during acquisition was stabilized using the Adaptative Focus module. GFP was excited at 488 nm and emitted light was collected at 500–550 nm. DiD was excited at 633 nm and the emission range was 640–700 nm. These imaging conditions ensured negligible bleed-through between GFP and DiD channels. Virus particles that entered the cell were tracked using the red channel as a reference. Real time single particle tracking analyses yielded the mean fluorescence intensity of viral particles for both red and green channels, and their coordinates, as a function of time. SVT analysis was performed using Imaris software (BitPlane, Switzerland).

Transmission electron microscopy

Cells were cultured on sterilized glass coverslips, fixed with 2.5% gluteraldehyde in 0.1 M Sodium Cacodylate Buffer at pH 7.2 or 0.1M PBS for 1 hr at RT then rinsed in sodium Cacodylate buffer 3 times, 15 mins each. Samples were stained in 1% osmium tetroxide in sodium Cacodylate buffer at RT for 1 h, rinsed with fresh Milli-Q water 3 times, 5 mins each, then stained with 2% uranyl acetate in Milli-Q water for 2 h at 4°C protected from light. The samples were rinsed with water for 10 mins, dehydrated in an ethanol series, embedded in resin and prepared for microscopy. Images were acquired on an FEI Tecnai 12 Transmission Electron Microscope

(TEM) with a 4 Megapixel Gatan Ultrascan™ 1000 CCD camera. Images were taken at 100-100,000x magnification using Gatan Digital Micrograph software at 120 kV using a lanthanum hexaboride (LaB6) electron source and analyzed using ImageJ software.

Confocal and STED microscopy image acquisition, processing and analysis

The microscope used for both confocal and STED images was based on an Abberior Instrument RESOLFT QUAD-P super-resolution microscope (Abberior Instruments) additionally equipped with two pulsed excitation lasers: 640 nm (LDH-D-C-640P, PicoQuant) and 485 nm (LDH-D-C-485P, PicoQuant), both with an 80 ps pulse width. The STED beam was produced by a titanium:sapphire laser system (MaiTai, Spectra-Physics) operating at 780 nm with a repetition rate of 80 MHz. The STED laser pulses were stretched to a pulse width of approximately 250-350 ps using two 20 cm SF6 optical glass rods, and a 121 m long polarization-maintaining single-mode optical fibre (OZ Optics). A phase-modifying plate (VPP-1a, RPC Photonics) was used in the STED beam path to generate a donut-shaped focal spot. STED and excitation laser beams were spatially superimposed and the fluorescence light was filtered using appropriate dichroic filters (AHF Analysentechnik). Precise positioning of the laser foci in the sample, and sample scanning was carried out using a QUAD beam scanner for lateral directions and an objective lens positioning system (MIPOS 100PL, Piezosystem Jena) for axial direction. The pulse timing was adjusted using a combination of an electronic delay box (Picosecond Delayer, Micro Photon Devices) and cables. Fluorescence excitation and collection used an oil immersion 100x, 1.40 NA objective (Olympus). The fluorescence signal was de-scanned, passed through an adjustable pinhole (Thorlabs) and detected by a single photon counting avalanche photo diode (SPCM-AQRH-13, Excelitas Techologies) with appropriate fluorescence filters. Data were acquired using Imspector™ software.

For confocal microscopy, images for each channel were acquired sequentially with following parameters: pinhole size: 1 Airy, *xy* pixel size: 100 nm, *z*-step size: 250 nm STED images were acquired sequentially for each channel with following parameters: pinhole size: 1 Airy, dwell time: 50 µs/pixel, *xy* pixel size: 40 nm, *z*-step size: 250 nm.

For STED microscopy images for each channel were acquired sequentially with following parameters: pinhole size: 1 Airy; dwell time: 50 µs/pixel, XY pixel size: 40 nm, Z-step size: 250 nm. STED images were deconvoluted using Huygens

Professional software using theoretical PSF parameters corresponding to the system's effective observation spot or point-spread-function (PSF, 50 nm in diameter, full-width-half-maximum of intensity profile) as confirmed by measurements of 20 nm fluorescent beads. All subsequent image manipulation steps were performed using Fiji (ImageJ distribution) software package. Minimal distance between HIV-1 particles and HFA plasma membrane was calculated using GFAP and HIV p24 fluorescent signals as guides. Due to the confocal only resolution in the *z* direction making accurate positioning difficult, distances were calculated in the *xy* plane only. Images in *z*-stack were analyzed separately under assumption that each virus particle is localized in a *z*-slice with the brightest p24 fluorescence signal. Images with a significant number of particles localized directly above or below the HFA membrane were omitted. Env content of HFA internalized and cell-free HIV-1 particles was calculated as follows. The percentage of Env^+ HIV-1 particles inside HFA was calculated by comparing the percentage of Env^+ HIV-1 particles inside HFA with cell free particles present within the same slide, using GFAP, p24 and gp120 fluorescence signals as guides.

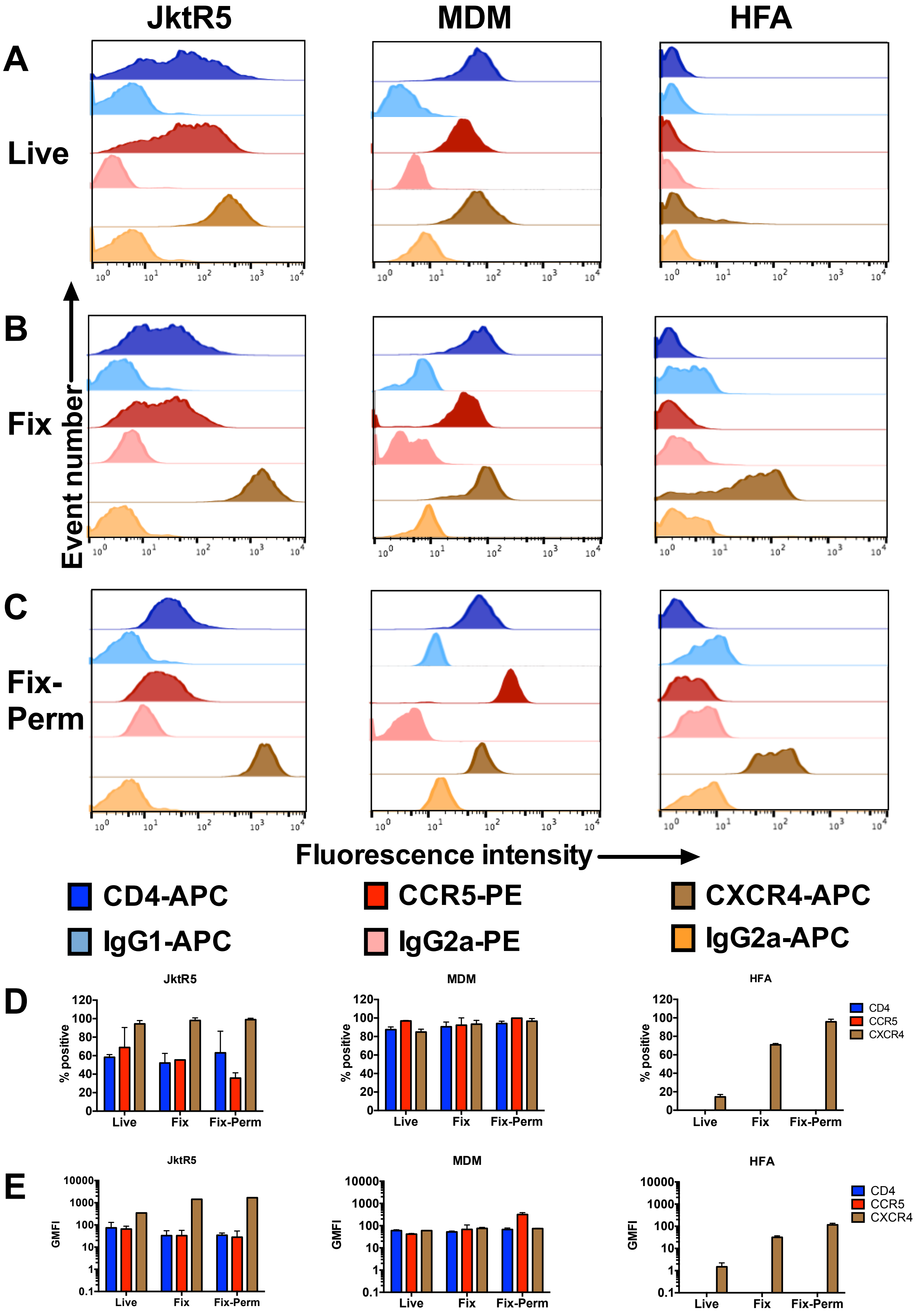
JktR5
MDM
HFA
A
Live
B
Fix
C
Fix-Perm
Event number
Fluorescence intensity
CD4-APC
IgG1-APC
CCR5-PE
IgG2a-PE
CXCR4-APC
IgG2a-APC
D
% positive
Live
Fix
Fix-Perm
CD4
CCR5
CXCR4
E
GMFI

Supplementary Figure 1. Flow cytometric analysis of HFA phenotype. A) Jurkat-R5 (Jkt-R5), monocyte-derived macrophages (MDM) and HFA were labeled live for CD4 (dark blue) or isotype control (light blue), CCR5 (red) or isotype control (pink) and CXCR4 (brown) or isotype control (orange) and analyzed by flow cytometry. **B)** Jkt-R5, MDM and HFA were fixed (Fix) and labeled as in **(A)**. **C)** Jkt-R5, MDM and HFA were fixed, permeabilized (Fix-Perm) and labeled as in **(A). D)** Summary of data from **(A-C)** showing % positive cells, n = 2 independent experiments with 2 independent macrophage donors. **E)** Summary of data from **(A-C)** showing geometric mean fluorescence intensity (GMFI) , n = 2 independent experiments with 2 independent macrophage donors.

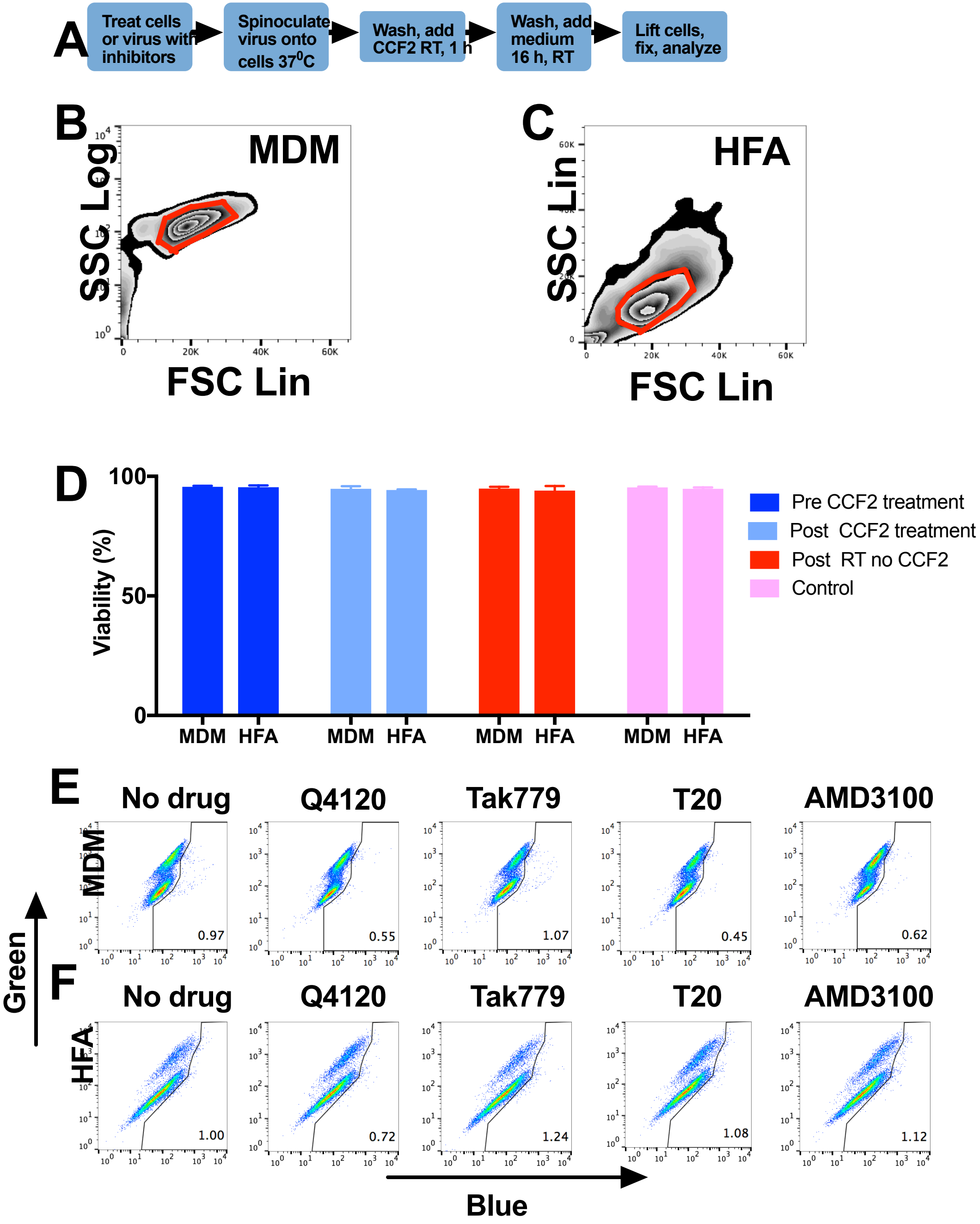

Supplementary Figure 2. HIV-1 LAI pseudotypes do not fuse with HFA, related to Figure 2. A) Experimental flow plan. **B)** Representative forward scatter (FSC) verses side scatter (SSC) of MDM after viral exposure and CCF2 treatment. Red region shows area selected for analysis in **E** and Figure 2. **C)** Representative FSC verses SSC of HFA after viral exposure and CCF2 treatment. Red region shows area selected for analysis in **F** and Figure 2. **D)** Cell viability before (Pre CCF2 treatment) and after CCF2 exposure (Post CCF2 treatment) compared to cells maintained at room temperature in the absence of CCF2 (Post RT no CCF2) and cells maintained at 37^{0}C with 5% CO_2 (Control), as determined by trypan blue, n = 1 independent experiment with technical triplicates. **E)** MDM were untreated or treated with inhibitors then transduced with LAI-pseudotyped BLaM-Vpr HIV-1 for 16 hr then cells lifted, fixed and analyzed by flow cytometry. **F)** HFA were untreated or treated with inhibitors then with LAI-pseudotyped BLaM-Vpr HIV-1 for 16 hr then cells lifted, fixed and analyzed by flow cytometry. See Figure 2H for quantified summary of data.

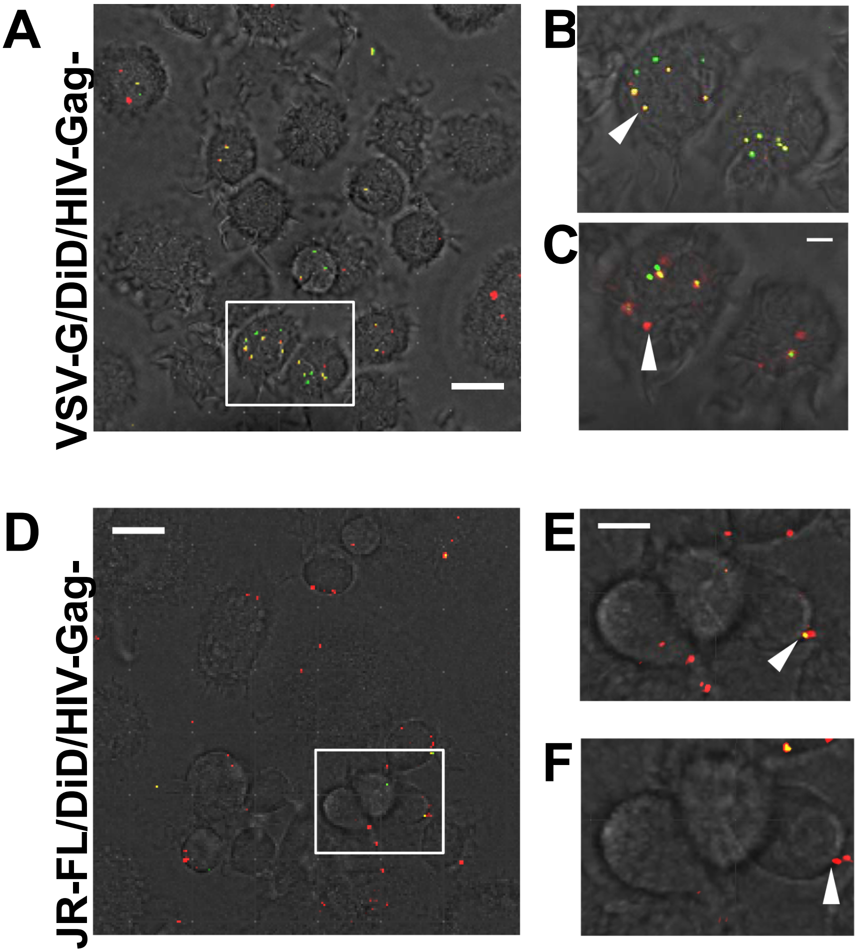

Supplementary Figure 3. Real-time single virus tracking reveals JR-FL and VSV-G-psuedotyped HIV-1 fusion with MDM, related to Figure 4. A) A representative experiment of particles double-labeled with DiD and Gag-iGFP, pseudotyped with VSV-G bound to live MDM. Scale bar 20 µm. **B** and **C)** Snap-shots of micrographs taken from the boxed section in **A** at 0 and 5 min, respectively. The yellow particle (white arrow, **B**) tracked over 5 min becomes red in **C** (white arrow). Scale bar: 2 µm. **D)** A representative experiment of particles double-labeled with DiD and Gag-iGFP, pseudotyped with JRFL bound to live MDM. Scale bar 20 µm. **E** and **F**) Snap-shots of micrographs taken from the boxed section in the **D** at 0 and 5 min, respectively. The yellow particle (white arrow, **E**) undergoes fusion and turns red (white arrow **F**). Scale bar: 4 µm. N = 3 independent experiments on 3 independent macrophage donors.

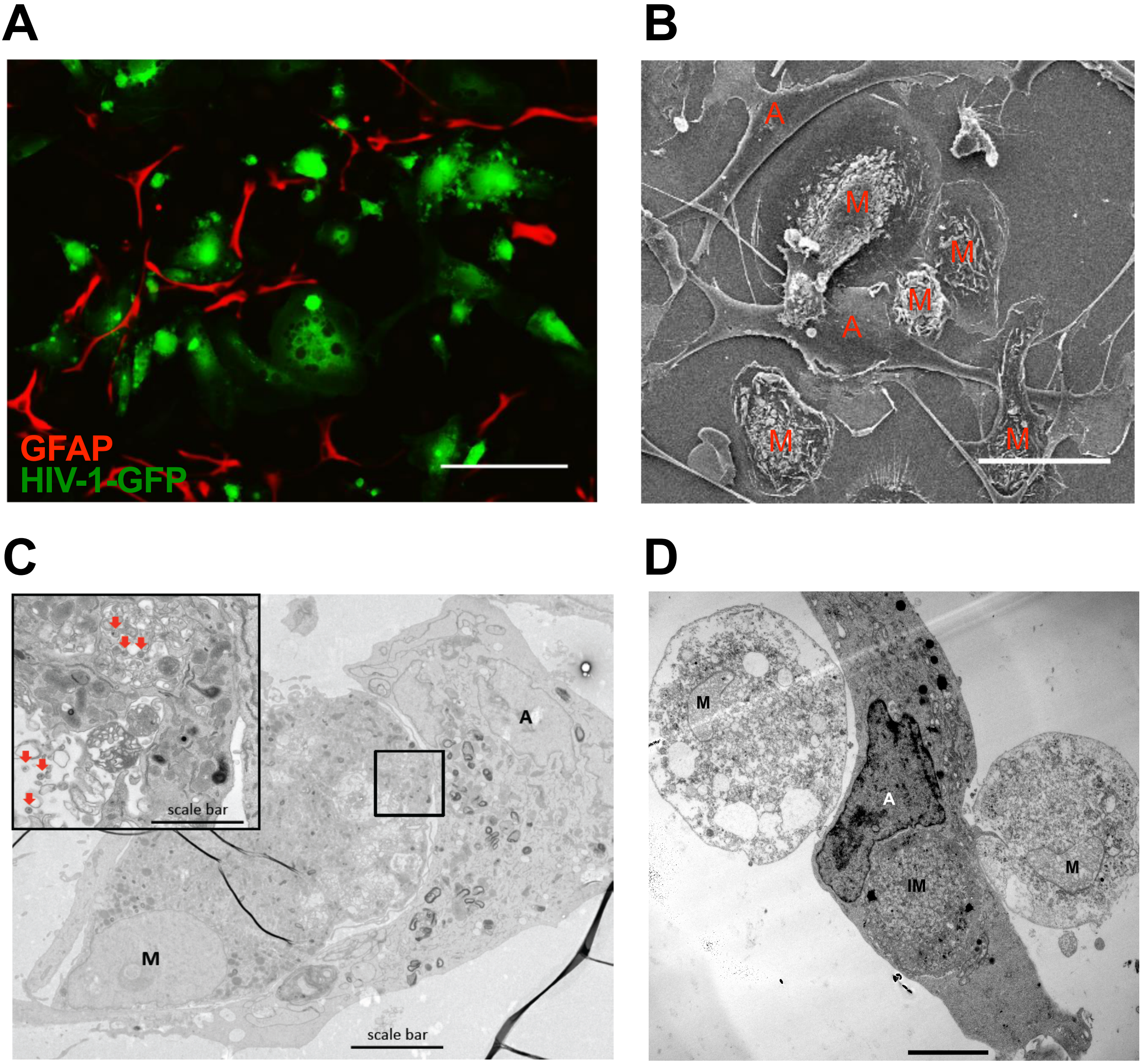

Supplementary Figure 4. Intimate associations form between HFA and HIV-1-infected MDM, related to Figures 5 and 6. A) Cocultures of HIV-$1_{JR\text{-}FL\text{-}GFP}$-infected MDM and GFAP-labeled HFA imaged by confocal microscopy, scale bar = 50 µm. **B)** SEM image of an MDM-HFA coculture showing MDM (M) interacting with HFA (A), scale bar = 25 µm. **C)** TEM image of HIV-1-infected MDM (M, labeled over nucleus) partially engulfed by an astrocyte (A. labeled over nucleus). Scale bar = 5 µm. Inset box shows higher magnification image, red arrows point to virions. Scale bar = 1 µm. **D)** TEM image of three MDM (M) interacting with HFA (A). IM = internalized macrophage. M and A label nuclei of cells. Scale bar = 5 µm

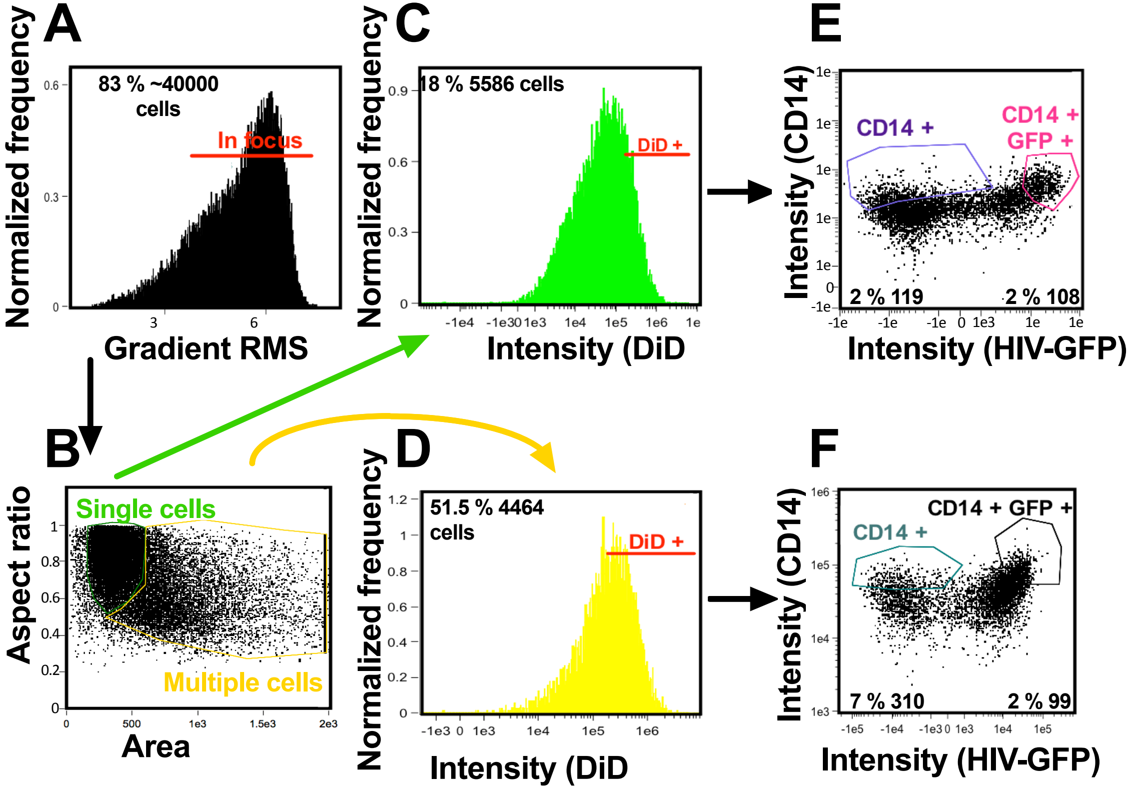

Supplementary Figure 5. Imagestream analysis of interactions between HFA and HIV-1-infected MDM, related to Figure 6. Gating strategy for image stream **A)** Selection of in focus cells. **B)** Selection of single and multiple cells. **C)** Selection of DiD^{+} single cells. **D)** Selection of DiD^{+} multiple cells. **E)** Selection of CD14^{+} and CD14^{+}/GFP^{+} cells within the DiD^{+} single cells. **F)** Selection of CD14^{+} and CD14^{+}/GFP^{+} cells within the DiD^{+} multiple cells.

Supplementary Movie 1. Real-time single virus tracking shows VSV-G-pseudotyped HIV-1 particles fusing with HFA related to Figure 4. VSV-G-psuedotyped HIV-1 virions double-labeled with Gag-iGFP (green signal) and DiD (red signal) were imaged in real time on live HFA plated in observation chambers under a confocal microscope. Double labeled virions (yellow) were tracked and fusion defined by the change from yellow to red (indicated by two white arrows). The movie plays at 12 frames / s and total duration is ~15 min.

Supplementary Movie 2. Real-time single virus tracking shows JRFL-pseudotyped HIV-1 particles fail to fuse with HFA, related to Figure 4. JRFL-pseudotyped HIV-1 virions double-labeled with Gag-iGFP (green signal) and DiD (red signal) were imaged in real time on live HFA plated in observation chambers under a confocal microscope. Double labeled virions (yellow) were tracked and failed to show fusion as defined by the change from yellow to red. Loss-of-focus and slight bleaching is observed in some of the particles over time. The movie plays at 12 frames / s and total duration is ~40 min.

Supplementary Movie 3. Real-time single virus tracking shows VSV-G-pseudotyped HIV-1 particles fuse with MDM, related to Figure S3. VSV-G-pseudotyped HIV-1 virions double-labeled with Gag-iGFP (green signal) and DiD (red signal) were imaged in real time on live MDM plated in observation chambers under a confocal microscope. A double labeled virion (yellow) was tracked and fusion defined by the change from yellow to red (indicated by white arrow). The movie plays at 12 frames / s and total and duration is ~7 min.

Supplementary Movie 4. Real-time single virus tracking shows JRFL-pseudotyped HIV-1 particles fuse with MDM, related to Figure S3. JRFL-pseudotyped HIV-1 virions double-labeled with Gag-iGFP (green signal) and DiD (red signal) were imaged in real time on live HFA plated in observation chambers under a confocal microscope. A double labeled virion (yellow) was tracked and fusion defined by the change from yellow to red (indicated by white arrow). The movie plays at 12 frames / s and total duration is ~10 min.

Supplementary Movie 5. Dynamic rendering of static images presented in Figure 6H, I.

Supplementary Movie 6. Dynamic rendering of static images presented in Figure 6J, K.
